# Flexible and cost-effective deep learning for accelerated multi-parametric relaxometry using phase-cycled bSSFP

**DOI:** 10.1038/s41598-025-88579-z

**Published:** 2025-02-09

**Authors:** Florian Birk, Lucas Mahler, Julius Steiglechner, Qi Wang, Klaus Scheffler, Rahel Heule

**Affiliations:** 1 Department of Biomedical Magnetic Resonance, University of Tübingen, Tübingen, Germany; 2 High-Field Magnetic Resonance, Max Planck Institute for Biological Cybernetics, Tübingen, Germany; 3 Center for MR Research, University Children’s Hospital, Zurich, Switzerland

**Keywords:** Phase-Cycled bSSFP, Deep Neural Networks, Multi-parametric Quantitative MRI, Relaxometry, MIRACLE, Magnetic resonance imaging, Learning algorithms

## Abstract

To accelerate the clinical adoption of quantitative magnetic resonance imaging (qMRI), frameworks are needed that not only allow for rapid acquisition, but also flexibility, cost efficiency, and high accuracy in parameter mapping. In this study, feed-forward deep neural network (DNN)- and iterative fitting-based frameworks are compared for multi-parametric (MP) relaxometry based on phase-cycled balanced steady-state free precession (pc-bSSFP) imaging. The performance of supervised DNNs (SVNN), self-supervised physics-informed DNNs (PINN), and an iterative fitting framework termed motion-insensitive rapid configuration relaxometry (MIRACLE) was evaluated in silico and in vivo in brain tissue of healthy subjects, including Monte Carlo sampling to simulate noise. DNNs were trained on three distinct in silico parameter distributions and at different signal-to-noise-ratios. The PINN framework, which incorporates physical knowledge into the training process, ensured more consistent inference and increased robustness to training data distribution compared to the SVNN. Furthermore, DNNs utilizing the full information of the underlying complex-valued MR data demonstrated ability to accelerate the data acquisition by a factor of 3. Whole-brain relaxometry using DNNs proved to be effective and adaptive, suggesting the potential for low-cost DNN retraining. This work emphasizes the advantages of in silico DNN MP-qMRI pipelines for rapid data generation and DNN training without extensive dictionary generation, long parameter inference times, or prolonged data acquisition, highlighting the flexible and rapid nature of lightweight machine learning applications for MP-qMRI.

## Introduction

Improving the efficiency and stability of quantitative magnetic resonance imaging (qMRI) methods is a crucial research task to enable clinical applicability, necessitating sophisticated acquisition, reconstruction, and postprocessing strategies. In addition to accurate morphological information, which MRI as a non-invasive imaging tool can provide to guide treatment^1^, qMRI has the potential to reduce subjectivity, resolve hardware or protocol dependencies inherent to conventional qualitative imaging, and increase intra- or interscanner reproducibility^2^, facilitating the decision-making process in the diagnosis and prognosis of diseases. Generally, qMRI aims at fitting multiple qualitative (weighted) images to quantitative parameter maps with a voxel-wise representation of biophysical and microstructural processes. The derived quantitative MR biomarkers, such as relaxometry metrics, offer great potential for early detection of pathological tissue changes or longitudinal monitoring of disease. Recent studies have shown that quantitative $T_2$ is an important marker of cortical pathology in multiple sclerosis patients^3,4^, early detection of hippocampal sclerosis in mesial temporal lobe epilepsy^5^, cerebrovascular disease^6^, or early Alzheimer’s disease^7,8^. Quantitative $T_1$ has proven beneficial for longitudinal studies to access microstructural changes related to brain aging^9^or Parkinson’s disease^10^. To reduce acquisition time, multi-parametric qMRI (MP-qMRI) has been of particular interest, aiming at the simultaneous estimation of multiple intrinsically co-registered parameter maps and a more complete neuroimaging protocol within feasible scan times^11,12^.

Jara et al.^12^ reported that MP-qMRI frameworks can be divided into direct and indirect frameworks. Direct MP-qMRI frameworks generate a series of data fragments, which are used for direct parameter interference^13–16^. Most popular amongst direct approaches is magnetic resonance fingerprinting (MRF)^13^, which was originally proposed for relaxometry and employs the acquisition of hundreds to thousands of data points using high undersampling factors to then quantify parameters of interest from the acquired tissue-specific signal evolutions. Thereby, MRF uses a pseudo-randomized pattern of continuously varying flip angles and repetition times, which is not necessarily efficient due to required relaxation delays to recover longitudinal magnetization. While earlier MRF implementations were adversely affected by prohibitively long combined image reconstruction and dictionary generation times^17^ or rather low resolution with thick slices to ensure sufficiently high signal-to-noise-ratios (SNRs)^18,19^, newer MRF techniques show promise for faster parameter interference^20^, more efficient data acquisition^21^, and application to diffusion quantification^22^ or magnetization transfer and chemical exchange imaging^23^.

Indirect MP-qMRI approaches, on the other hand, rely on clinically interpretable fully reconstructed weighted images for post-hoc mapping of parameters of interest^24–29^. Steady-state free precession (SSFP) sequences such as phase-cycled balanced SSFP (pc-bSSFP)^27,28^ or multi-pathway non-balanced SSFP^26^ are popular choices for indirect MP-qMRI relaxometry since they exhibit a pronounced mixed $T_1$ and $T_2$sensitivity and allow an efficient acquisition of multiple contrasts without the need for extensive undersampling, waiting times, or long reconstruction times, while providing isotropic whole-brain coverage. Furthermore, similar to MRF, SSFP imaging is versatile as it can be sensitized to various biochemical and microstructural properties beyond relaxometry, such as magnetization transfer^30–32^, diffusion^33,34^, or electrical conductivity^35^.

Machine learning (ML) techniques, in particular deep neural networks (DNNs), have shown great success for both direct and indirect MP-qMRI frameworks. Dictionary generation and matching in the case of MRF^19,20,36,37^ or multi-parametric inference from multi-contrast SSFP data^38,39^ can substantially be accelerated using DNNs. Data-driven model-free methods that leverage measured input and ground truth data for supervised learning are capable of eliminating the estimation bias due to oversimplified existing signal models, for example as a result of unaccounted microstructural features as in the case of single-component simultaneous $T_1$ and $T_2$quantification based on pc-bSSFP^38^. The primary constraints of in vivo supervised learning are the dependence of the trained DNN on specific measurement protocols, time-consuming acquisition of ground truth data, limited hardware and data accessibility, and unknown model assumptions as part of black-box modeling. In silico data generation, on the other hand, allows maximum control over the training data used and is becoming increasingly important in the (pre)training of ML models. It was reported that DNNs trained on in silico data for MP-qMRI are influenced by the chosen training data distribution^40^. Gyori et al. showed that selecting a uniform or an in vivo data distribution for the target parameters of interest differently affects the precision and accuracy of supervised DNN predictions. Recent research has compared supervised deep neural networks with physics-informed self-supervised decoding-encoding deep neural networks in the context of joint diffusion and $T_1$ quantification^41^.

This study proposes the use of in silico pc-bSSFP data to train DNN models as flexible and cost-effective frameworks for multi-parameter estimation. To this end, we compare three methods for in vivo whole-brain MP-qMRI relaxometry targeted on the simultaneous estimation of $T_1$ and $T_2$ in tissue, including a supervised DNN (SVNN), a physics-informed self-supervised DNN (PINN), and a conventional relaxometry method called motion-insensitive rapid configuration relaxometry (MIRACLE)^27^ as reference. We investigate the impact of training data distribution on the reliability of the parameter estimation for both DNN methods. The robustness of the trained SVNNs and PINNs as well as conventional MIRACLE in the presence of noise-corrupted data is analyzed based on a Monte Carlo (MC) estimation of accuracy and precision metrics. To demonstrate the adaptability of the proposed DNNs, we implement an extended DNN, which effectively includes phase information of the acquired MR data into the training process to allow accelerated data sampling suited for application in clinical settings where acquisition speed is crucial. Ultimately, we evaluate the flexibility of DNNs in learning the inverse signal model for parameter estimation in terms of convergence speed during training and estimation speed of the final DNN models.

## Methods

The following subsections describe the in vivo data acquisition and processing, the in silico signal generation, the DNN and MIRACLE frameworks for relaxometry fitting, and the experiments to validate the in silico and in vivo performance. Lastly, as clinical use case an adapted DNN for accelerated data acquisition is presented. All in vivo experiments were conducted at a field strength of 3 T (Magnetom Prisma, Siemens Healthineers, Erlangen, Germany) and in accordance with the guidelines of the ethics committee of the Faculty of Medicine at the Eberhard Karls University of Tübingen. Three healthy subjects without any known prior medical conditions were included in this study. Python and PyTorch were used for data simulation, data processing, as well as DNN training and fitting.

### Data acquisition in vivo

For in vivo validation, sagittal 3D pc-bSSFP data were used, acquired in three healthy subjects with a $N_{pc} = 12$ phase-cycling scheme using radiofrequency (RF) phase increments $\phi$ evenly distributed in the range 0 to ${2\pi }: \phi (j) = \pi /N_{pc}\cdot (2j-1)$, where j = 1, 2, ...$N_{pc}$. The bSSFP imaging protocol employed an isotropic resolution of 1.3 x 1.3 x 1.3 mm$^{3}$ with an image encoding matrix of 176 × 176 × 128, ensuring coverage of the entire brain. The repetition time (TR) and echo time (TE) were set to 4.8 ms and 2.4 ms, respectively, and the nominal flip angle $\alpha _{nom}$ was fixed at 15$^\circ$. Prior to the acquisition of each phase cycle $\phi$, 256 dummy pulses were played out to establish steady-state conditions. Incorporating a 2-fold in-plane parallel imaging (Generalized Autocalibrating Partial Parallel Acquisition (GRAPPA)) acceleration factor, the acquisition of whole-brain 12-point pc-bSSFP data was completed within 10 min 12 s. To illustrate the potential for clinical applicability, scans with $N_{pc} = 6$ and 4 phase cycles were conducted in one healthy subject, resulting in acquisition times of 5 min 6 s and 3 min 24 s, respectively. The $B_1^+$ scaling factor ($\alpha _{act}/\alpha _{nom}$ = actual/nominal flip angle) was calculated employing the vendor’s standard $B_1^+$ mapping sequence^42,43^, including a TR/TE/$\alpha _{nom}$ of 14.2 s/2.4 ms/8$^\circ$, 30 sagittal slices with a 100% slice gap, an in-plane resolution of 2.4 x 2.4 mm$^{2}$, a slice thickness of 3 mm, and a total scan time of 29 s.

### Data processing in vivo

Registration and segmentation tasks were performed using the FSL^44^ and SPM^45^ software packages. To correct for motion, intra-registration along the phase cycle dimension was achieved by registering the magnitude of each bSSFP phase cycle to the magnitude of the middle, i.e. ($N_{pc}/2$)th, phase cycle and applying each transformation to the corresponding phase data. Constant receiver-related phase offsets were removed by subtracting the average phase across phase cycles. In case of multiple phase-cycled bSSFP scans with varying number of phase cycles, the scans with 6 and 4 phase cycles were co-registered to the scan with 12 phase cycles. In addition, rigid body registration was used to align the $B_1^+$ baseline anatomical image to the mean magnitude image from the motion-corrected pc-bSSFP data. The obtained transformation was applied to the $B_1^+$ map, which was then 3D median filtered ($\text {kernel size} = \left[ 10, 10, 10\right]$).

The bSSFP signal can be written as a Fourier series with coefficients $F_n$ (also referred to as SSFP configurations or SSFP modes), which reflect free induction decay (FID)-like ($F_n$, $n\ge 0$) and echo-like ($F_n,$ $n<0$) signal contributions^46–48^ with distinct $T_1$ and $T_2$ sensitivity. These configurations can be isolated based on a discrete Fourier transform of the acquired series of bSSFP images with varying RF phase increments (phase cycles)^49^and can then be utilized for relaxometry^27^. To avoid aliasing in the $F_n$ modes, the number of acquired phase cycles needs to be sufficiently high. Aliasing due to the finite sampling of phase cycles introduces an off-resonance sensitivity for low $N_{pc}$, which leads to a modulation of the magnitude and phase of the $F_n$ modes (cf. Supplementary Fig. S1). Therefore, correct modeling of the extracted $F_n$ modes in the presence of aliasing, i.e. in practice in case of a low number of acquired phase cycles, necessitates consideration of off-resonance effects (see section below). It has further to be noted that the signal level of the $F_n$ configurations decays relatively rapidly with higher orders^50^. Here, the three lowest-order SSFP configurations $F_{-1}$, $F_0$, and $F_1$ (cf. Supplementary Fig. S1) were computed based on a discrete Fourier transform of the acquired complex pc-bSSFP data. The magnitude and phase of the retrieved SSFP configurations were further subjected to Gibbs ringing removal^51^. Voxel-wise normalization using Euclidean distance was performed to match the in silico data.

For in vivo SNR determination, the average signal level was obtained as the mean signal in a whole-brain tissue mask applied to the magnitude of the $F_0$ configuration, pooled across three representative subjects. The average noise level was determined as the mean standard deviation in a background mask applied to the same data. The average SNR level pooled across all three subjects was 25. The definition of the masks for the in vivo SNR determination is illustrated in Supplementary Figure S2.

### Data generation *in silico*

Synthetic single isochromat pc-bSSFP signals $S_{\text {bSSFP}}$ were generated using the forward bSSFP signal model $S_{\text {bSSFP}}(\text {p},\text {u})$ with parameters $\text {p} \in \left\{ T_{1}, T_{2}, B_1^+, \Delta {B_0}\right\}$ (longitudinal relaxation time $T_1$; transverse relaxation time $T_2$; transmit field scaling factor $B_1^+ = \alpha _{act}/\alpha _{nom}$; off-resonance due to local variations in the static field $\Delta {B_0}=\theta /(2\pi \cdot TR)$, with $\theta$ referring to the off-resonance-related phase accumulation within TR), sequence parameters $\text {u} \in \left\{ \text {TR}, \text {TE}, \alpha _{nom}, N_{pc}\right\}$ (repetition time TR; echo time TE; nominal flip angle $\alpha _{nom}$; number of phase cycles $N_{pc}$), and initial magnetization $M_0 = 1$^49^:

1 $$\begin{aligned} S_{\text {bSSFP}} = M_0 \frac{(1-E_1)(1-E_2e^{-i\psi })\sin {\alpha _{act}}}{C\cos {\psi }+D} e^{-\text {TE}/T_2} e^{i\theta (TE/TR)} \end{aligned}$$

with

$$\begin{aligned} & \psi = \theta - \phi \\ & E_{1,2} = e^{-\text {TR}/T_{1,2}} \\ & C = E_2(E_1 -1)(1+\cos {\alpha _{act}}) \\ & D = (1-E_1\cos {\alpha _{act}})-(E_1 - \cos {\alpha _{act}})E_{2}^2 \end{aligned}$$

and

$$\begin{aligned} \phi [0, 2\pi ]=\pi /N_{pc}\cdot (2j-1), j = 1,2, ...,N_{pc} \end{aligned}$$

**Fig. 1:**
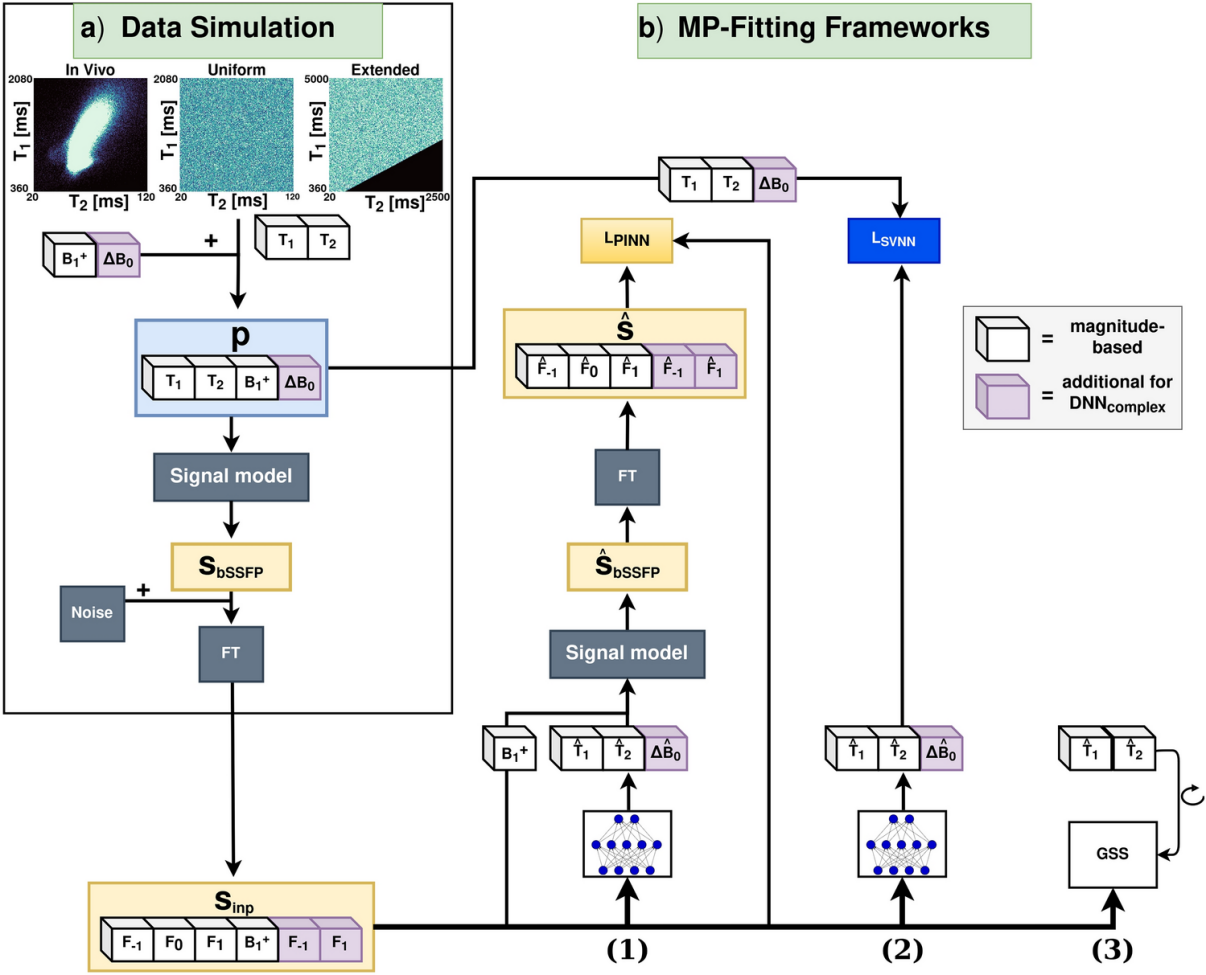
The workflow proposed in this work (purple cubes represent the extended input and output in case of complex-based DNNs). (**a**) **Data Simulation:** The input parameters $\text {p} = \left\{ T_{1}, T_{2}, B_1^+, \Delta B_0 \right\}$ entering the analytical bSSFP signal model (see Eq. 1) were sampled from three different distributions (in vivo, uniform, and uniform extended) for $T_1$ and $T_2$, and from a single uniform distribution for $B_1^+$ and $\Delta B_0$. The sequence parameters from the in vivo acquisition protocol (TR, TE, $\alpha _{nom}$, $N_{pc}$) were used to draw 400,000 signal samples $S_{\text {bSSFP}}$ from each $T_1$ and $T_2$ distribution. (**b**) **Multi-Parametric-Fitting Frameworks:** The input to each of the three frameworks, which means the physics-informed neural network (PINN or $PINN_{complex},$ 1), the supervised neural network (SVNN or $SVNN_{complex},$ 2), and the iterative golden section search (GSS) fitting (MIRACLE, 3), consisted of the amplitudes (magnitude-based) or real and imaginary parts (complex-based, without imaginary part of $F_0$) of the three lowest-order SSFP configurations computed from a Fourier transform (FT) of the phase-cycled bSSFP signal with the option to add noise and in addition of $B_1^+.$ 1) and 2) use the same multilayer perceptron architecture (magnitude-based: 64 neurons per hidden layer, complex-based: 256 neurons per hidden layer) to estimate the inverse signal model and predict the parameters $\hat{\text {p}} \in \left\{ \hat{T}_{1}, \hat{T}_{2}, \hat{\Delta {B}}_0\right\}$. 1) uses the predicted $\hat{T}_1$, $\hat{T}_2$, and $\hat{\Delta B_0}$ (with the addition of the $B_1^+$ input) to generate an estimated signal $\hat{S}$ and compare it to the input signal $S_{\text {inp}}$ in the $L_{\text {PINN}}$ loss, while 2) compares the predicted $\hat{T}_1$, $\hat{T}_2$, and $\hat{\Delta B_0}$ directly to the respective ground truth target parameters $\text {p} \in \left\{ T_{1}, T_{2}, \Delta {B_0}\right\}$ in the $L_{\text {SVNN}}$ loss. The off-resonance $\Delta {B_0}$ was only utilized for the complex-based DNNs (purple cubes).

The target parameters $T_1$ and $T_2$ were sampled from three different distributions (see Fig. 1a): a uniform distribution with $T_1$ ranging from 360 to 2080 ms and $T_2$ ranging from 20 to 120 ms, a uniform distribution with an extended $T_1$ range from 360 to 5000 ms and an extended $T_2$ range from 20 to 2500 ms, and an in vivo distribution with the same range as the uniform distribution, but by sampling from a 2D density map generated based on the $T_1$ and $T_2$ brain voxel distributions of three healthy subjects obtained from existing gold standard 2D multi-slice inversion-recovery turbo-spin-echo ($T_1$) and 2D multi-slice single-echo spin-echo ($T_2$) scans with variable inversion and echo times, respectively. Corresponding anatomical magnetization-prepared rapid gradient-echo (MPRAGE) data^52^ were skull-stripped and used for white matter (WM), gray matter (GM), and cerebrospinal fluid (CSF) segmentation. Voxels containing pure CSF according to the performed segmentation were excluded from the density estimation. The $T_1$ and $T_2$ parameter boundaries of the in vivo and uniform distribution were approximated as the mean ± 2 standard deviations of the values in the defined brain masks of three subjects. Additionally, $T_1 < T_2$ parameter combinations were excluded for all distributions. For each distribution, 400,000 samples were generated, resulting in a total training data size of 38.4 MB and 6.4 MB for the input and target data, respectively. $B_1^+$ was uniformly sampled in the range 0.7 to 1.3.

Two different DNN training strategies were investigated: a standard magnitude-based DNN for direct comparison with MIRACLE using magnitude $F_n$ input data and an extended complex-based DNN utilizing the full information of the complex-valued $F_n$ data as input (identified throughout this article by the subscript *complex*, e.g. $DNN_{complex}$) to allow a reduction of the number of phase cycles. For the standard magnitude-based DNNs, an on-resonant condition with an off-resonance $\Delta B_0 = 0$ Hz was assumed for the in silico data generation. For the complex-based DNNs, the off-resonance-related phase accumulation $\theta$ was uniformly sampled in the range $-\pi$ to $+\pi$ without including the boundaries at $\pm \pi$ to deliberately exclude in silico training data near phase wraps, thereby enhancing the accuracy of $\Delta B_0$ estimation and facilitating efficient training of the complex-based DNNs.

In silico sequence parameters in Eq. 1 were set according to the in vivo pc-bSSFP protocol with a matched number of phase cycles. For in silico performance validation, an additional 2D grid (200 x 200 steps) of linearly sampled $T_1$ and $T_2$ values in the uniform distribution range as well as 40,000 in vivo test data points sampled from the 2D in vivo density map were generated for pc-bSSFP signal simulation ($B_1^+ = 1$).

### Relaxometry

For direct comparison, the standard magnitude-based DNNs were designed to take the same input data as MIRACLE, i.e. the magnitude of the three lowest-order SSFP configurations ($F_{-1}$, $F_0$, $F_1$) and $B_1^+$, as shown in Fig. 1. To account for aliasing in accelerated data with a reduced number of phase cycles and to improve robustness to off-resonance, the standard DNNs were extended to take the real part of $F_{-1}$, $F_0$, $F_1$, the imaginary part of $F_{-1}$ and $F_1$ as well as $B_1^+$ as input ($DNN_{complex}$, extended input represented as purple cubes in Fig. 1). The imaginary part of $F_{0}$ was zero due to the employed subtraction of the average phase across phase cycles and was thus omitted. MIRACLE fitting was performed using an iterative golden section search minimization algorithm with an initial $T_1$estimate of 1000 ms^26,27^. The standard magnitude-based DNNs were based on a fully connected feed-forward multilayer perceptron with four input neurons, two hidden layers of 64 neurons each, followed by a ReLU activation function, and an output sigmoid layer of two neurons for $T_1$ and $T_2$ estimation. The complex-based DNNs were based on the same training architecture, but extended to utilize six input neurons and three output neurons for $T_1$, $T_2$, and $\Delta {B_0}$ estimation, with an increased number of 256 neurons in the two hidden layers. The resulting models contained 4610 and 68355 trainable parameters with a total size of 21kB and 276kB for the standard magnitude-based and complex-based DNNs, respectively.

The trainable parameters were initialized using PyTorch’s default layer initialization^53^ and the Adam optimizer^54^. A fixed learning rate of $2\cdot 10^{-4}$, a batch size of 128, an early stopping with a patience of 25 epochs, and a maximum of 300 epochs were used for all DNN trainings. Within each training batch, the real and imaginary parts of the pc-bSSFP data were corrupted by additive Gaussian noise samples with a noise level of $\eta = 0.074/(\sqrt{2}\cdot \text {SNR})$ and $\text {SNR} \in \left\{ \text {inf}, 50, 25, 10 \right\}$, where $\eta$ is zero if $\text {SNR} = \text {inf}$. The three lowest-order SSFP configurations were computed as described above in the subsection *Data Processing In Vivo*. The DNN frameworks were designed to decode the inverse signal model from the pc-bSSFP signals to target relaxometry parameters by employing two different loss strategies as proposed by Grussu et al.^41^: a signal loss $L_{\text {PINN}} = \text {MSE}(\hat{S},S_{inp})$ involving the analytical pc-bSSFP signal model and subsequent Fourier transform of the complex signal in the encoding step to compute the mean squared error (MSE) between the signal from the predicted target parameters $\hat{S}$ and the input signal $S_{inp}$ (cf. Fig. 1b, part 1), and a target parameter loss $L_{\text {SVNN}} = \text {MSE}(\hat{{\textbf {p}}},{\textbf {p}})$, which computes the MSE between the model parameter predictions $\hat{\text {p}} \in \left\{ \hat{T}_{1}, \hat{T}_{2}, \hat{\Delta {B}}_0\right\}$ and the ground truth target parameters $\text {p} \in \left\{ T_{1}, T_{2}, \Delta {B_0}\right\}$ (cf. Fig. 1b, part 2). The off-resonance $\Delta {B_0}$ was only utilized for the complex-based DNNs (cf. purple cubes in Fig. 1).

### Validation of magnitude-based DNNs

#### In silico

The standard magnitude-based DNN frameworks, trained with different distributions and SNR levels, as well as the MIRACLE framework were validated on 5000 MC samples by augmenting the complex pc-bSSFP signals from the 2D linear grid and in vivo distribution test data of $T_1$ and $T_2$ values with additive noise from a Gaussian distribution and $\text {SNR} \in \left\{ \text {inf}, 50, 45, 40, 35, 30, 25, 20, 15, 10 \right\}$. To test the accuracy and precision of each framework on in silico data, the mean $\mu _{\text {MC}}$, standard deviation $\sigma _{\text {MC}}$, and relative standard deviation $\sigma _{rel}=\sigma _{\text {MC}}/\mu _{\text {MC}}$ of the parameter predictions across all MC samples were calculated. The relative error between the MC mean of the parameter predictions $\hat{y}_{\mu _{\text {MC}}}$ and the respective ground truth value *y* was calculated for both DNN frameworks on the 2D linear $T_1$ and $T_2$ sampling grid as $\epsilon _{rel} = (\hat{y}_{\mu _{\text {MC}}}-y)/y\cdot 100$. In addition, the coefficient of determination (CoD) was calculated for all frameworks, distributions, and SNR levels for the entire 2D grid and the in vivo test data. The CoD was computed as a global metric as follows:

2 $$\begin{aligned} \text {CoD} = 1 - \frac{\sum _{i=1}^{n}(y_i - \hat{y}_{i_{\mu _{\text {MC}}}})^2}{\sum _{i=1}^{n}(y_i -\bar{y})^2} \end{aligned}$$

where:

$$\begin{aligned}&n \text { is the number of observations,} \\&y_i \text { is the observed value for the ith observation,} \\&\hat{y}_{i_{\mu _{\text {MC}}}} \text { is the MC mean of the predicted values for the ith observation,} \\&\bar{y} \text { is the mean of the observed values.} \end{aligned}$$

#### In vivo

For the standard magnitude-based DNN and MIRACLE relaxometry frameworks, simultaneous whole-brain $T_1$ and $T_2$ estimation was performed in a healthy test subject. SVNN and PINN frameworks trained without the addition of noise during training (SNR = inf) were used for in vivo inference. To compare the effect of different training data distributions on prediction accuracy, the absolute difference between the DNN predictions from the trainings with three distributions and the MIRACLE prediction was calculated ($\Delta T_i = \hat{T}_{i, DNN} - \hat{T}_{i, MIRACLE}$ with i = 1, 2). In addition, MC sampling was performed on an exemplary axial slice of the in vivo data with 5000 samples and six augmented ROIs with additional Gaussian noise added to the real and imaginary parts of the acquired pc-bSSFP data before calculating the SSFP configurations.

### Complex-based DNNs for accelerated data acquisition

As a direct result from the observed influence of training data distribution and noise characteristics on the performance of magnitude-based DNNs, complex-based DNNs were trained on noise-free training samples with the uniform $T_1$ and $T_2$ distribution. The training was repeated using in silico training data with 12, 6, and 4 bSSFP phase cycles. The performance of the derived complex-based DNNs was validated in silico and in vivo versus magnitude-based DNNs trained on identical training data and versus MIRACLE applied to a reduced number of phase cycles. In silico, the accuracy was assessed as the relative error between the parameter predictions $\hat{T}_i$ and the simulated ground truth value $T_i$: $\epsilon _{rel}(T_i) = (\hat{T}_i-T_i)/T_i\cdot 100$ with i = 1, 2. In vivo, the parameter estimations with 12 phase cycles of the respective relaxometry method of interest ($\hat{T}_{i, 12 pc}$) served as reference. The absolute difference between the parameter predictions with 6 and 4 phase cycles ($\hat{T}_i$) relative to the reference with 12 phase cycles was calculated as $\Delta T_i = \hat{T}_i - \hat{T}_{i, 12 pc}$ with i = 1, 2.

### Flexible and cost-effective relaxometry

The effectiveness of the magnitude- and complex-based DNN frameworks to learn the inverse signal model for MP-qMRI was investigated by calculating the CoD between whole-brain relaxometry predictions of each epoch and the final epoch of a training process for whole-brain WM, GM, and WM+GM tissue masks. Furthermore, a single-epoch DNN training was performed and applied to the in vivo test subject. The entire process of in silico data generation, single-epoch learning, and in vivo inference was timed and compared to the MIRACLE algorithm on whole-brain pc-bSSFP data (single CPU thread on Intel(R) Xeon(R) W-2255 CPU @ 3.70GHz, 62.5 GB RAM). To assess the benefit of the trained DNN frameworks, the inference time for simultaneous in vivo whole-brain relaxometry was additionally measured for all three frameworks on input data interpolated to different isotropic resolutions of 1.3 mm, 1.0 mm, 0.8 mm, 0.6 mm, and 0.4 mm.

## Results

### Validation of magnitude-based DNNs

#### In silico

**Fig. 2:**
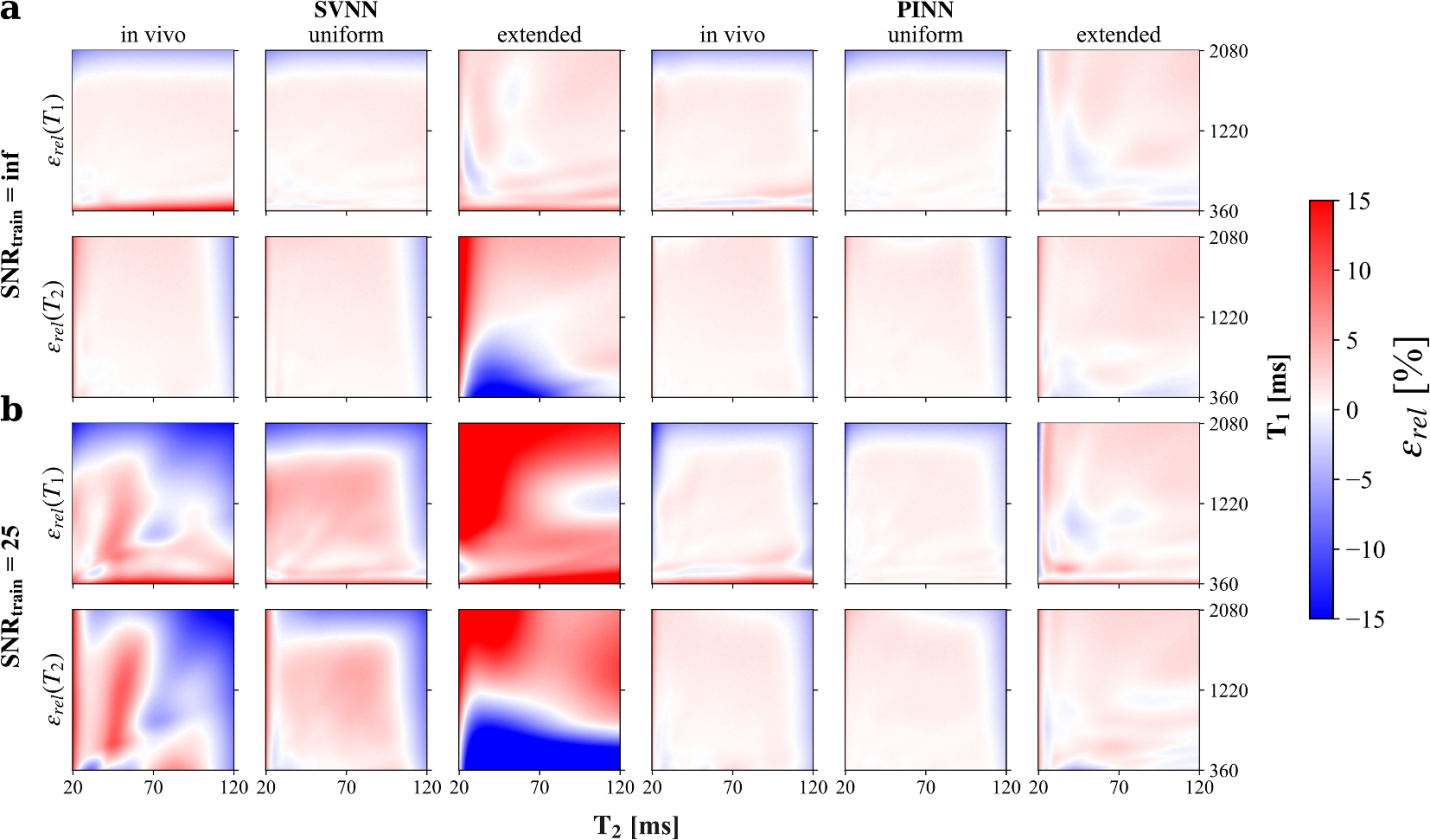
Influence of training data SNR and training data distribution on accuracy of investigated DNNs in silico. The relative error in percent $\epsilon _{rel} (T_i) = (\hat{T}_{i_{\mu _{\text {MC}}}}-T_i)/T_i\cdot 100$ with $i = 1,2$, between the mean of the MC simulation $\hat{T}_{i_{\mu _{\text {MC}}}}$ and the ground truth $T_i$, is quantified for $T_1$ and $T_2$ parameter estimates of the SVNNs (left) and PINNs (right) trained on noise-free (**a**, SNR = inf) and noise-corrupted (**b**, SNR = 25) data with different training data distributions. The MC estimation is performed on a noise-corrupted in silico linear test grid with $\text {SNR} = 25$ matched to in vivo conditions as well as a $T_1$ and $T_2$ range corresponding to brain tissues (consistent with the parameter range of the in vivo and uniform distribution employed for DNN training). Parameter over- and underestimation with respect to the ground truth are shown in red and blue, respectively.

The impact of including image noise explicitly into the DNN training process by adding noise of a predefined level to the in silico training data is analyzed by an MC sampling of the in silico test data for DNNs trained on three different training data distributions (cf. Fig. 2). To ensure comparability with the acquired in vivo data, the noise added during training was matched to the SNR of 25 present in the masked brain tissue of the in vivo pc-bSSFP data and applied to the test data. As evident from Fig. 2, training DNN frameworks under non-ideal conditions with noise-corrupted training samples does not imply better accuracy on test data with equal SNR level. While the accuracy of PINNs trained on noise-corrupted data (Fig. 2b, right) appears similar to that of PINNs trained on noise-free data (Figure 2a, right), SVNNs perform worse when training includes noise (Fig. 2, left). Furthermore, it can be observed that the performance of the trained PINN models is largely independent of the training data distribution, in contrast to the SVNN frameworks. In the case of the uniform distribution with extended parameter range, the SVNN shows reduced accuracy compared to the other two distributions. Since training with additional noise evidently does not improve prediction performance, the following analysis focuses on the application of DNNs trained on noise-free in silico data. Therefore, the indication of SNR or noise levels refers in the following exclusively to the test data rather than the training data.

**Fig. 3:**
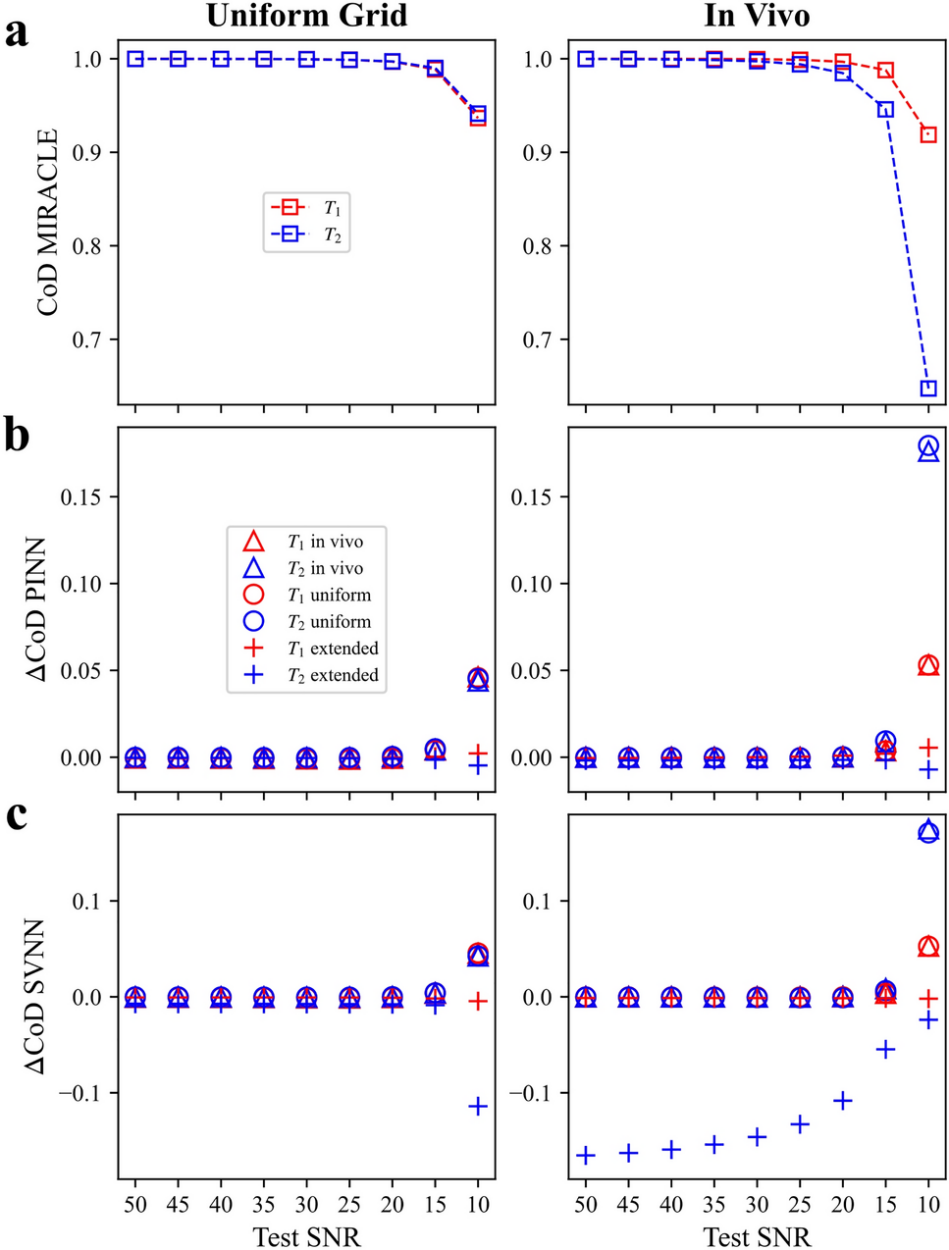
Coefficient of determination versus test data SNR of investigated DNNs relative to MIRACLE in silico. The CoD between the mean MC relaxation parameter predictions $\hat{T}_{i_{\mu _{\text {MC}}}}$ and the ground truth $T_{i}$ with $i = 1,2$ ($T_1$ in red and $T_2$ in blue) is shown for the linear test grid (left column) and the in vivo distribution test data (right column). (**a**) CoD versus test data SNR for MIRACLE ($\square$). (**b**) and (**c**) The absolute CoD difference between the DNNs and MIRACLE ($\Delta \text {CoD} = \text {CoD}_{\text {DNN}}-\text {CoD}_{\text {MIRACLE}}$) versus test data SNR for the PINN (**b**) and the SVNN (**c**). For both SVNN and PINN, three models trained on noise-free data (SNR = inf) with different data distributions are evaluated: in vivo ($\triangle$), uniform ($\circ$), and uniform extended distribution ($+$). Note that positive/negative values in (**b**) and (**c**) are referring to higher/lower CoD values of the DNNs relative to MIRACLE.

The prediction performance dependence of the DNNs trained with noise-free data and MIRACLE on the SNR of the in silico test data is evaluated in Fig. 3 by the calculation of the CoD, reflecting the agreement between the mean MC predictions and the ground truth for test data from a linear sampling grid (cf. Fig. 3, left column) and from the in vivo distribution (cf. Fig. 3, right column). High CoD values can be observed for MIRACLE at SNR levels down to $\approx 15-20$ until the accuracy starts to break down (cf. Fig. 3a). While the difference between the CoD of the DNNs and MIRACLE ($\Delta \text {CoD}$) is neglectable for high test SNR levels, the performance of the DNNs trained with the uniform and in vivo distributions is superior to MIRACLE at low SNR levels ($\le 15$) (cf. Fig. 3b and c). The DNNs trained with the uniform extended distribution show reduced CoD values on noisy test data comparable to MIRACLE. Only exception is the SVNN-based $T_2$ estimation, which shows a clearly lower accuracy than MIRACLE for the in vivo distribution test data in case of the uniform extended training data distribution (cf. Fig. 3c, right). The robustness and performance advantage over MIRACLE of the DNNs trained with data distributions matched to the in vivo tissue $T_1$ and $T_2$ range, i.e. uniform and in vivo distributions, is further corroborated by a precision analysis on in silico data (cf. Supplementary Fig. S3, test data SNR = 25) and is in particular evident in low SNR scenarios (cf. Supplementary Fig. S4, test data SNR = 10), while the uniform extended distribution demonstrates similar precision as MIRACLE.

####  In vivo

**Fig. 4:**
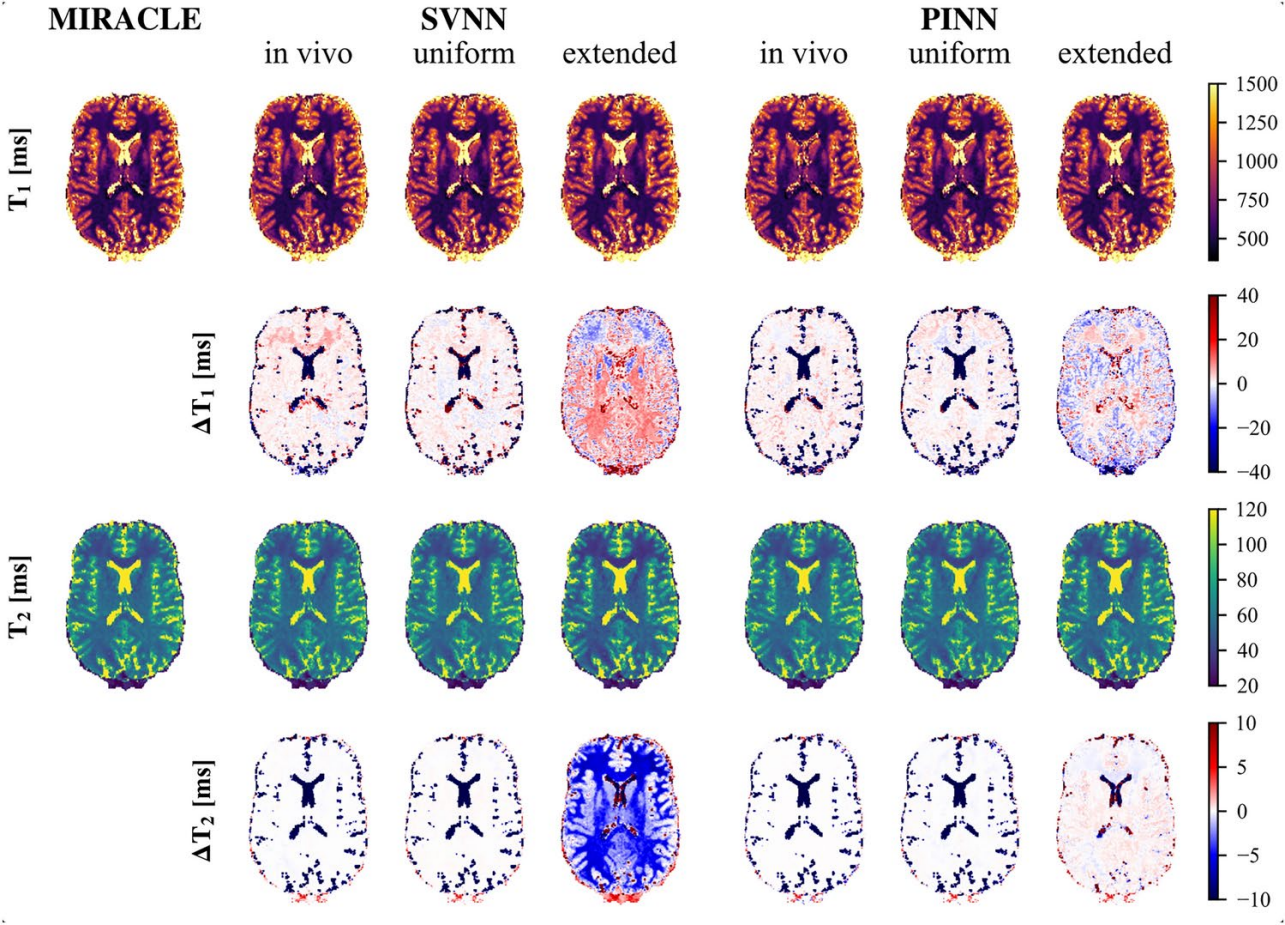
In vivo analysis of the effect of the DNN training data distribution relative to MIRACLE. A representative axial slice of the in vivo whole-brain $T_1$ (first row) and $T_2$ (third row) predictions of an unseen test subject is shown for the MIRACLE framework (first column), and both DNNs, each trained on in silico data without additional noise (SNR = inf) and three different distributions (in vivo, uniform, and uniform extended). The absolute differences ($\Delta T_i = \hat{T}_{i, DNN} - \hat{T}_{i, MIRACLE}$ with i = 1, 2) between the DNN predictions and the MIRACLE prediction are shown in the second and fourth row for $T_1$ and $T_2$, respectively. Red and blue refer to an over- and underestimation of the DNN framework predictions relative to the MIRACLE framework predictions, respectively.

In line with the in silico results in Figs. 2 and 3, the trained DNNs show high agreement with MIRACLE relaxometry in brain tissues when tested on unseen in vivo data, especially for the uniform and in vivo training parameter distributions, which are optimized for brain tissue at the employed field strength (cf. Fig. 4). The relaxation parameter values predicted by the SVNN framework, which was trained with the uniform extended distribution and thus for a parameter range covering not only relaxation times in tissues but also in fluids, deviate from $T_1$ and $T_2$ provided by MIRACLE. On the other hand, the PINN framework shows greater robustness to the underlying training data distribution with lower differences to the MIRACLE predictions, especially for $T_2$, but also $T_1$. Furthermore, the in vivo MC sampling demonstrates an increased precision (lower $\sigma _{MC}$) for DNNs trained with the uniform and in vivo distributions as compared to MIRACLE, while the uniform extended distribution performs similarly, in accordance with the in silico results (cf. Supplementary Figs. S5, S6, and S7).

### Complex-based DNNs for accelerated data acquisition

#### In silico

**Fig. 5:**
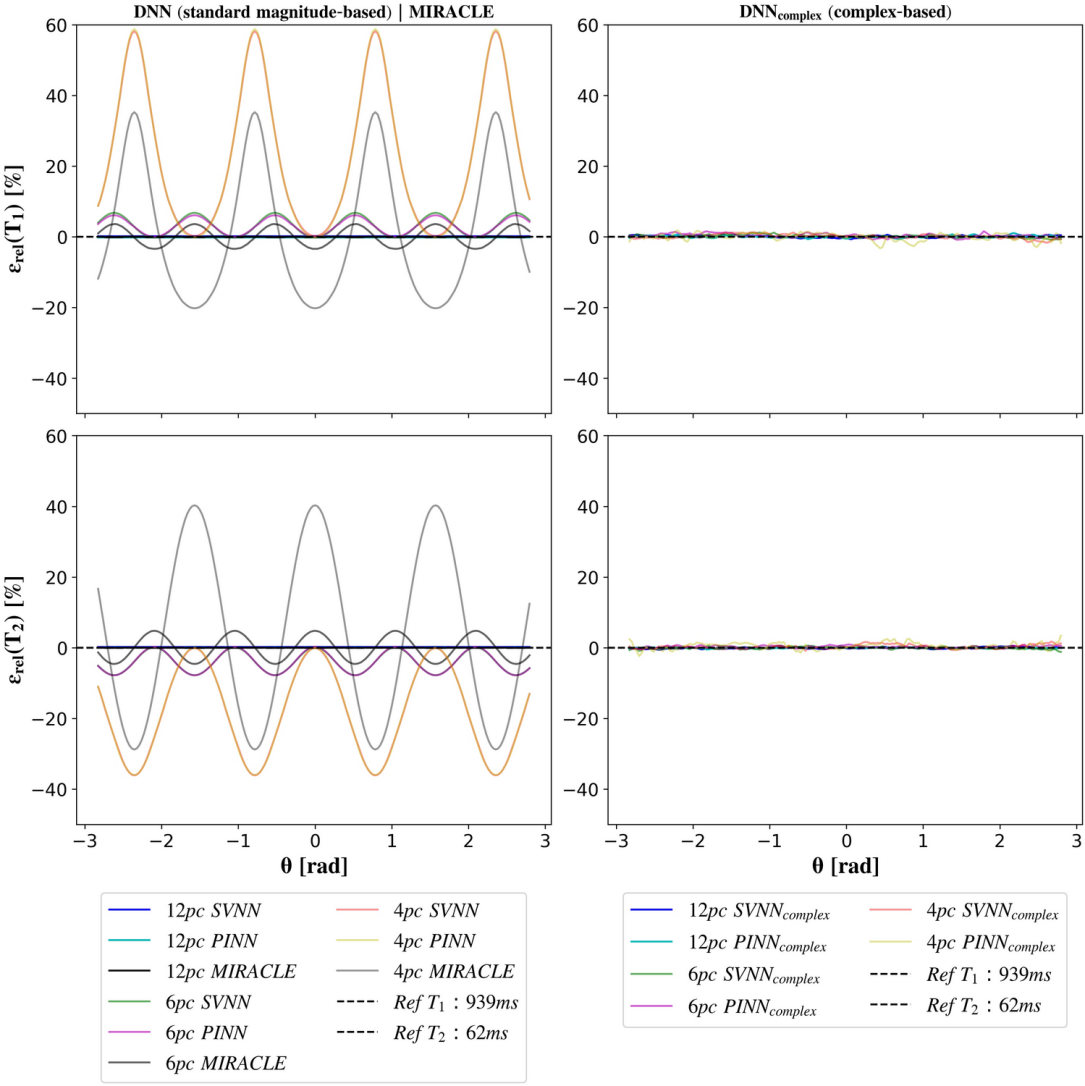
Influence of off-resonance-related phase accumulation within TR ($\theta$) on DNN and MIRACLE relaxometry in silico depending on the number of bSSFP phase cycles. DNNs were trained on noise-free data from a uniform distribution of $T_1$ and $T_2$ and for a varying number of phase cycles ($N_{pc} \in \left\{ 12, 6, 4 \right\}$). The noise-free test data were simulated based on reference $T_1$ (62 ms) and $T_2$ (939 ms) white matter relaxation values at 3 T^55^. The relative error between the parameter predictions $\hat{T}_i$ and the simulated ground truth value $T_i$, $\epsilon _{rel}(T_i) = (\hat{T}_i-T_i)/T_i\cdot 100$ with i = 1, 2, is shown for MIRACLE and the standard magnitude-based DNNs (left column) as well as the complex-based DNNs (right column). Dashed lines indicate the 0 $\%$ error for reference.

The in silico results presented in Fig. 5 demonstrate robust parameter prediction and successful elimination of off-resonance sensitivity of the proposed complex-based DNNs (right column) compared to the magnitude-based DNN and MIRACLE frameworks (left column). The aliasing introduced into the three lowest-order SSFP configurations in case of a limited number of only 6 or 4 phase cycles and the resulting errors in parameter predictions are effectively mitigated by utilizing the full information of the complex-valued SSFP modes as input, increasing the number of neurons per hidden layer, and incorporating $\Delta B_0$ as an additional target parameter. Both $SVNN_{complex}$ and $PINN_{complex}$ reliably estimate $T_1$ and $T_2$ for noise-free test data across the entire range of simulated $\Delta B_0$ values, achieving overall low relative errors of below $1\%$, $1.5\%$, and $3.5\%$ for 12, 6, and 4 phase cycles, respectively.

#### In vivo

**Fig. 6:**
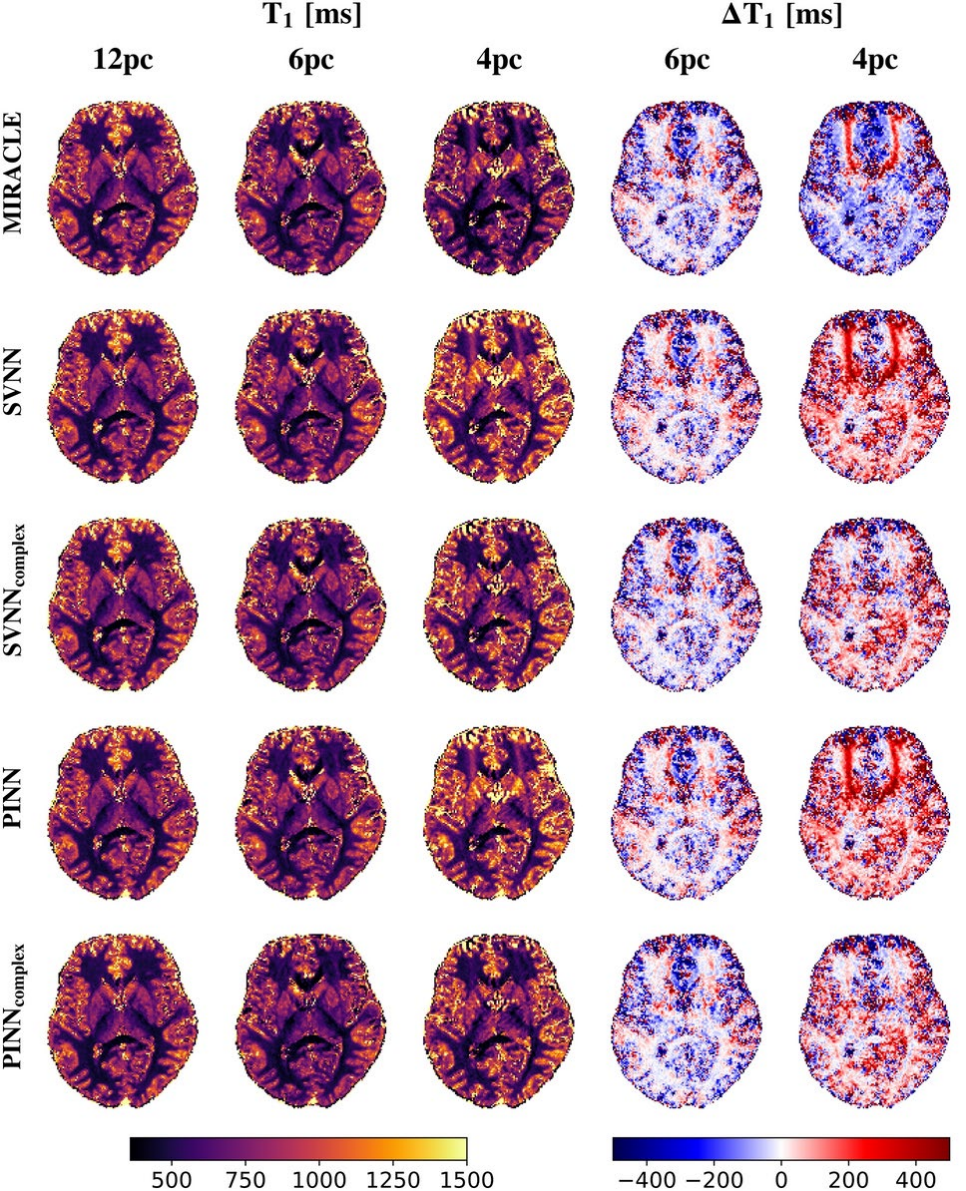
Performance of complex-based DNN versus MIRACLE and magnitude-based DNN in vivo $T_1$ estimation in case of accelerated pc-bSSFP acquisitions with only 6 and 4 phase cycles in comparison to the standard protocol with 12 phase cycles. A representative axial slice of the in vivo whole-brain $T_1$ predictions of an unseen test subject obtained with 12, 6, and 4 phase cycles is shown in the first, second, and third column for each framework (MIRACLE, SVNN, $SVNN_{complex}$, PINN, $PINN_{complex}$, from top to bottom), respectively. The absolute differences $\Delta T_1 = \hat{T}_1 - \hat{T}_{1, 12 pc}$ between the parameter predictions with 6 and 4 phase cycles relative to the reference with 12 phase cycles are shown in the fourth and fifth column, respectively.

**Fig. 7:**
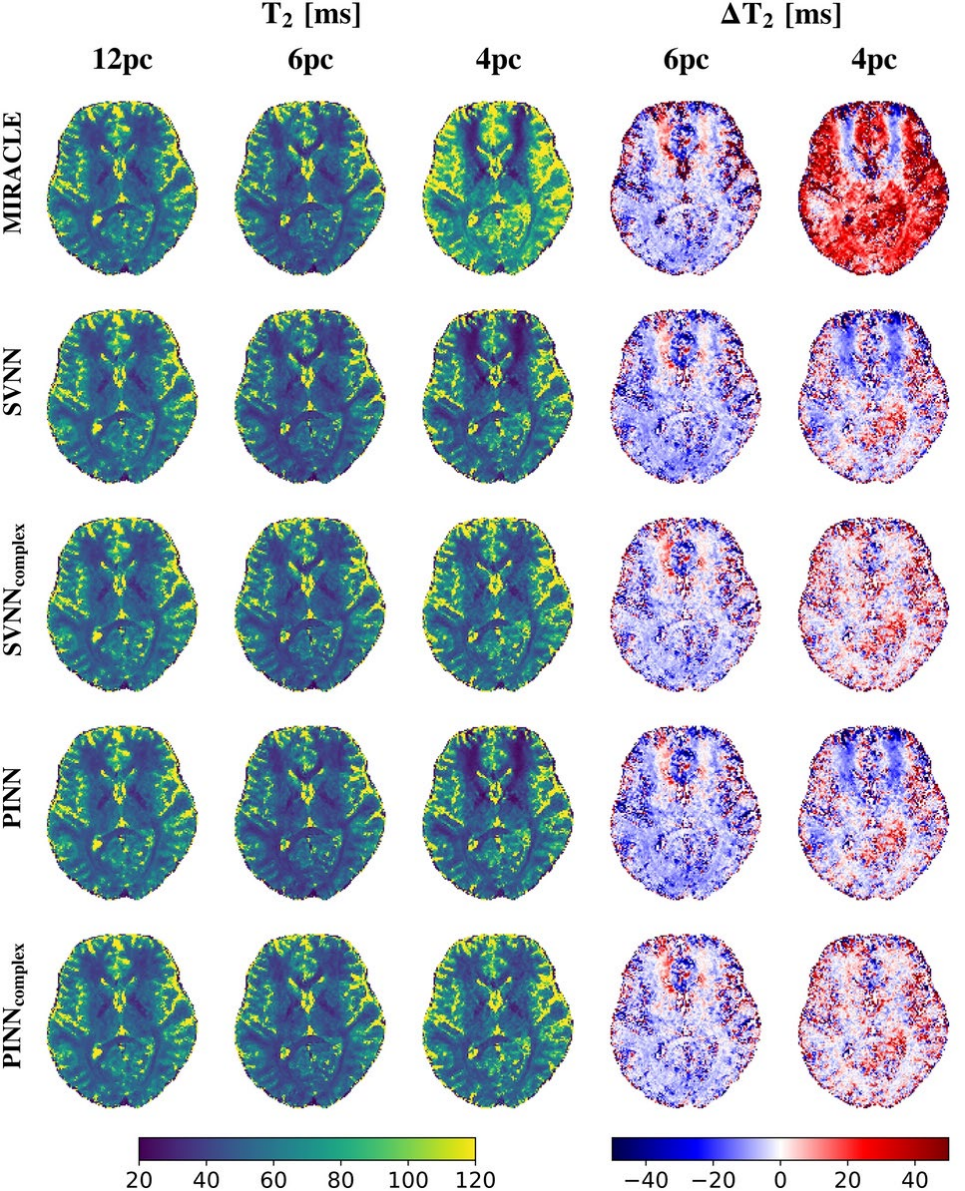
Performance of complex-based DNN versus MIRACLE and magnitude-based DNN in vivo $T_2$ estimation in case of accelerated pc-bSSFP acquisitions with only 6 and 4 phase cycles in comparison to the standard protocol with 12 phase cycles. A representative axial slice of the in vivo whole-brain $T_2$ predictions of an unseen test subject obtained with 12, 6, and 4 phase cycles is shown in the first, second, and third column for each framework (MIRACLE, SVNN, $SVNN_{complex}$, PINN, $PINN_{complex}$, from top to bottom), respectively. The absolute differences $\Delta T_2 = \hat{T}_2 - \hat{T}_{2, 12 pc}$ between the parameter predictions with 6 and 4 phase cycles relative to the reference with 12 phase cycles are shown in the fourth and fifth column, respectively.

Consistent with the in silico results presented in Fig. 5, the trained complex-based DNNs demonstrate enhanced robustness to off-resonances for in vivo $T_1$ (cf. Fig. 6) and $T_2$ (cf. Fig. 7) relaxometry, in particular for a low number of phase cycles. At 12 phase cycles, the $T_1$ and $T_2$ maps of all investigated relaxometry frameworks appear very similar, while considerable differences arise in case the number of phase cycles is reduced to 4. In that case, both MIRACLE and standard magnitude-based DNNs show distinct regional patterns of pronounced under- and overestimations in $T_1$ and $T_2$ due to off-resonance sensitivity, in particular in frontal brain regions close to the sinuses reflecting the underlying inhomogeneities in $B_0$ in those regions. Furthermore, MIRACLE tends to systematically underestimate $T_1$ (cf. Fig. 6) and overestimate $T_2$ (cf. Fig. 7) globally in brain tissue for $N_{pc}=4$. The magnitude-based DNNs exhibit a tendency to systematically overestimate $T_1$ for $N_{pc}=4$, while no general systematic bias in the $T_2$ estimates can be observed, but off-resonance-related artifacts persist. The complex-based DNNs achieve further improvement and effectively eliminate off-resonance-related artifacts and related systematic biases, especially at 4 phase cycles. Also in case of 6 phase cycles, the complex-based DNNs appear still superior to MIRACLE and magnitude-based DNNs with reduced absolute differences relative to the 12 phase cycle reference, e.g. in global WM.

### Flexible and cost-effective relaxometry

**Fig. 8:**
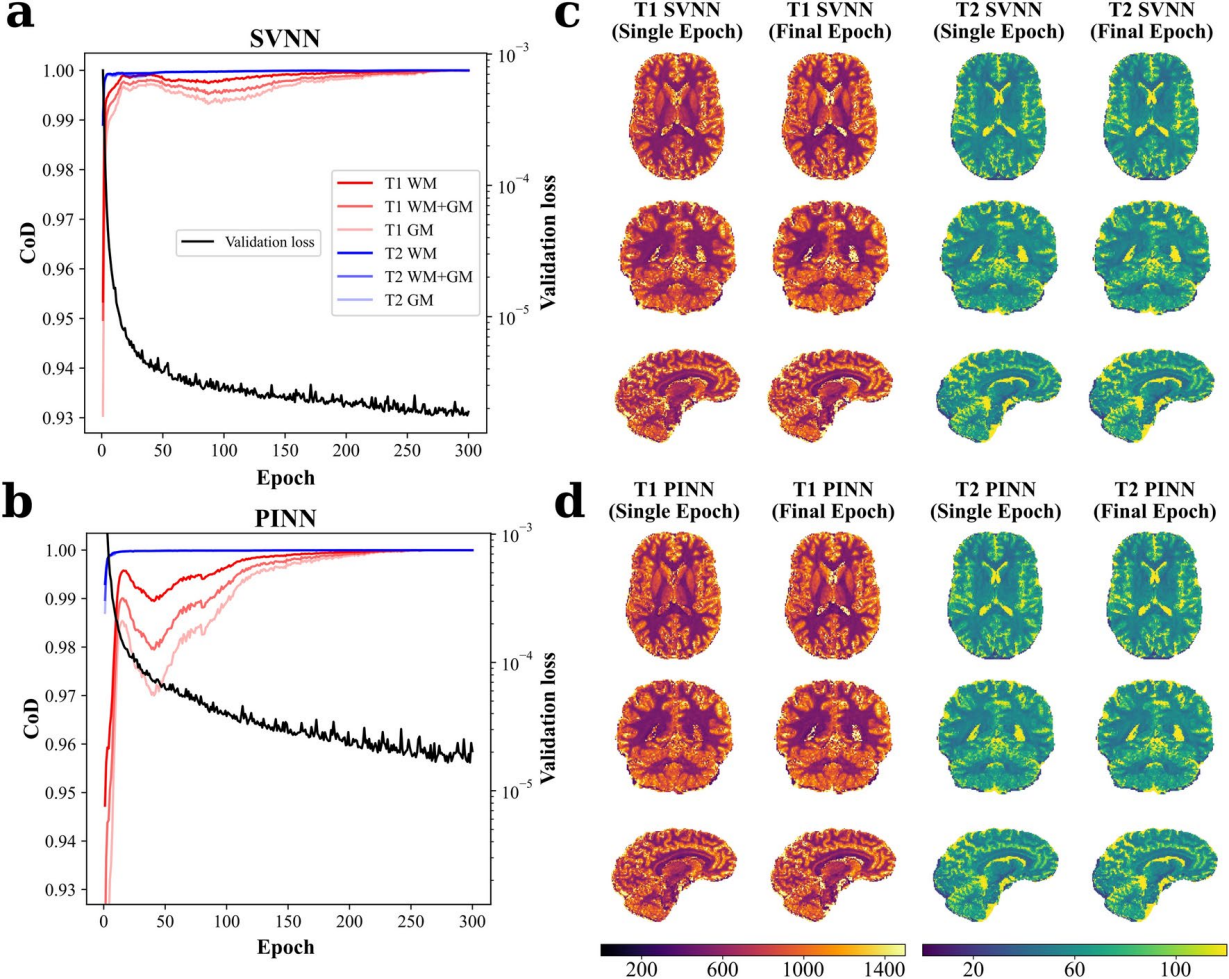
Efficiency of standard magnitude-based DNN inverse signal model learning versus epochs, corroborated by representative relaxation time maps of single-epoch in vivo whole-brain inference. The CoD during SVNN (**a**) and PINN (**b**) training is calculated for each epoch with respect to the final-epoch model and plotted versus epochs for in vivo $T_1$ (red) and $T_2$ (blue) predictions in whole-brain WM, GM, and WM+GM tissue masks of an unseen test subject. Additionally, the validation loss for both DNN frameworks is shown in black on a logarithmic scale. The employed DNNs were trained on the in silico uniform noise-free data distribution. Note that the final validation loss of the SVNN framework is on the order of one magnitude lower than the one of the PINN framework due to the different definitions of the loss functions and embedding of physical constraints for the PINN. Corresponding representative axial, coronal, and sagittal slices of in vivo whole-brain $T_1$ and $T_2$ single-echo versus final-epoch predictions of an unseen test subject are shown for SVNN (**c**) and PINN (**d**). See Supplementary Figure S8 for the corresponding results with complex-based DNNs.

The adaptability and flexibility of in silico DNN training is evaluated in Fig. 8a (SVNN) and Fig. 8b (PINN) by the CoD between the in vivo predictions of each epoch and the final epoch in different whole-brain tissue masks as well as the overall validation loss across epochs, representative for a DNN training using the uniform data distribution. Final training convergence with a CoD > 0.99 was reached after about 200 epochs. However, already within the first epochs, $T_1$ and $T_2$ relaxation times of brain tissues were learned effectively. This is corroborated by CoD values higher than 0.93 and 0.88 after the very first, and higher than 0.99 and 0.97 after the first ten epochs for the SVNN (cf. Fig. 8a) and PINN (cf. Fig. 8b), respectively, across all investigated tissue masks. Increasing the number of trainable parameters for the complex-based DNNs resulted in a higher number of epochs required to reach CoD > 0.9 in case of $T_1$ estimation with $PINN_{complex}$ (cf. Supplementary Fig. S8). Training of a single epoch was completed after only about 9s for the SVNN (10s for $SVNN_{complex}$) and 14s for the PINN (20s for $PINN_{complex}$) frameworks using a single CPU thread. The effectiveness of single-epoch versus final-epoch training is demonstrated for in vivo whole-brain relaxometry in Fig. 8c and d for the SVNN and PINN frameworks, respectively. The entire process of training data simulation, single-epoch model training, and whole-brain in vivo inference at 1.3 mm isotropic resolution took only about 12 s and 17 s for the standard magnitude-based SVNN and PINN frameworks, respectively, thus only about 45 % and 64 % compared to the inference time of the MIRACLE algorithm applied to the same data by using the same computing power.

Another advantage of using DNNs over the MIRACLE framework for simultaneous whole-brain relaxometry is the inference time of the final trained models, as demonstrated in Supplementary Figure S9. When the resolution of the in vivo input data is increased from 1.3 mm to 0.8 mm or even to 0.4 mm isotropic voxel sizes, the inference time increases drastically from 26 s to 94 s to 753 s for MIRACLE, compared to 2 s to 8 s to 65 s for the PINN (and similarly for the SVNN) and 9 s to 36 s to 287 s for the $PINN_{complex}$ (and similarly for the $SVNN_{complex}$). The inference time using the standard magnitude-based DNNs is thus always in the order of one magnitude ($\approx$ factor 3 for complex-based DNNs) lower, allowing fast parameter estimation for very high-resolution whole-brain data in only about 10% (about 30% for complex-based DNNs) of the inference time compared to MIRACLE.

## Discussion

The optimization of DNN model architectures for parametric mapping may require adaptation to altered tissue parameter characteristics, for example at different field strengths as in case of relaxation times, or to altered scan parameters at sites with different MR vendors, resulting in the time-consuming and thus costly requirements for additional in vivo measurements or re-running of DNN training. Therefore, flexible and effective DNN frameworks are a necessity. The results presented in this work suggest the combination of in silico trained DNNs with pc-bSSFP imaging as a fast and adaptable framework for MP-qMRI relaxometry. In silico DNN training allows full control over sequence parameters and tissue parameter distributions, does not require any extra measurements of ground truth data, and is able to efficiently learn the inverse signal model. Since the training process is purely based on simulations, it can easily be matched to optimized in vivo protocols and flexibly be adapted to altered sequence parameters such as TR or flip angle at a minimal cost. This approach further offers the option to include a range of repetition times and flip angles in the simulation of the training data to cover a range of different acquisition protocols at once, which could be investigated in future.

Both supervised and self-supervised physics-informed DNNs were successfully implemented and trained on different in silico data distributions, achieving a performance matching or exceeding the one of reference iterative multi-parametric fitting approaches such as MIRACLE with whole-brain in vivo inference times on unseen test data, which were up to an order of magnitude shorter in comparison to MIRACLE. Not only a considerably faster parameter inference could be achieved, but the investigated complex-based DNNs also yielded a clear performance advantage over conventional MIRACLE in case of accelerated data acquisition by undersampling along the phase cycle dimension, enabling reliable whole-brain relaxometry at isotropic high resolution in scan times as short as 3.4 min.

MC simulations based on in silico data (cf. Figure 2) revealed a strong sensitivity of SVNN estimation accuracy to the training data distribution, but also to the SNR level of the training data while the PINN models remained highly unaffected by the distribution and noise characteristics of the training samples. This makes PINN models more adaptable for scenarios with different $T_1$ and $T_2$ characteristics or field map distributions, which may not be known a priori, for example for the translation to different field strengths, to different anatomical targets, or to pathological data.

Generally, the DNNs trained on noise-corrupted training data and tested on data at the same SNR level did not reveal any ability to improve $T_1$ and $T_2$ prediction performance as compared to DNNs trained without any additional noise. In case of the SVNNs, adding noise levels matched with the observed in vivo SNR to the training samples even resulted in worse performance. Based on this finding, this work focused on the training of DNNs on noise-free data. Increasing the complexity of DNNs may allow to capture the noise present in the training data. However, sample-wise noise addition may be unrealistic for the spatially varying noise characteristics encountered in reconstructed MR images and hinder efficient learning of the signal model, especially for smaller DNN architectures with fewer trainable parameters. Provided the accessibility of larger cohort data sets, image-based DNNs could be investigated in future for denoising tasks.

The DNNs trained on noise-free in silico data with a uniform parameter distribution matched to the relaxation time range of tissues or an in vivo parameter distribution performed reliably in the presence of noise on the test data, with an advantage over MIRACLE for low SNR scenarios (cf. Fig. 3), which may be particularly beneficial for potential future applications at low-fields ($B_0 \le {1.5}{T}$). In addition, for distributions tailored to the relaxation time range of interest, DNNs show the ability to reach higher precision than MIRACLE, motivating the optimization of DNN frameworks for targeted tissue parameter ranges. The lower the SNR of the input data, the more pronounced becomes the precision advantage of the DNNs over MIRACLE (cf. Supplementary Fig. S3 and S4).

The in silico results were successfully reproduced on in vivo test data, revealing a stronger dependence on the training data distribution of the SVNN framework compared to the PINN framework (cf. Fig. 4). The observed influence of training data distribution on the accuracy of SVNNs is consistent with existing research^40,56^. Epstein et al. proposed to adjust the in silico ground truth labels by precomputed labels from maximum likelihood estimation and to extend the supervised loss to improve the accuracy of SVNNs^56^. On the other hand, the observed robustness of PINNs to the underlying training data distributions can be explained by a successful utilization of the analytical pc-bSSFP signal model, which encodes the estimated parameters into the pc-bSSFP signal during the learning process. Additionally, we observed that the DNNs, which were trained on data distributions optimized for the brain tissue parameter range, achieved lower standard deviations in the in vivo MC simulations for added noise levels and thus increased precision compared to MIRACLE (cf. Supplementary Fig. S5, S6, and S7), in line with the in silico findings.

Inherent to the architecture of PINNs are the boundaries of the achievable parameter values, which are predefined by the developer, prohibiting extrapolation. Thus, a limitation of PINNs is the need for retraining if the parameter range of interest falls outside the simulated range. Similarly, the performance of SVNNs is expected to be impaired for parameter combinations not contained in the training set. This applies not only to the range of $T_1$ and $T_2$ values, but also to the $\Delta B_0$ and $B_1^+$ distributions. In case of $\Delta B_0$, the training samples of the complex-based DNNs essentially covered a $2\pi$ range for the off-resonance-related phase accumulation ($\theta$), excluding the boundaries at $\pm \pi$ to aid better convergence of the complex-based DNNs. Since the signal of the complex-valued $F_n$ modes is $2\pi$-periodic in $\theta$ after removal of constant phase offsets along the phase cycle dimension (cf. Supplementary Fig. S1), the simulated range of $\theta$ is sufficient to ensure reliable $T_1$ and $T_2$ estimation of $\Delta B_0$ even outside the $2\pi$ range. If reliable $\Delta B_0$ estimation was anticipated, which was not the target of this work, unwrapping strategies would be required to account for $2\pi$ phase wraps. In case of $B_1^+$, the trained DNNs yield accurate relaxometry values for $B_1^+$ values in the range from 0.7 to 1.3, which corresponds to the range of the in silico training data (cf. Supplementary Fig. S10). However, it has to be noted that the performance depends on the accuracy of the external $B_1^+$ derivation. If there is a mismatch between the measured and the actual $B_1^+$, the $T_1$ estimation becomes biased. Interestingly, the $T_2$ estimation remains largely unaffected as long as the actual $B_1^+$ lies within the range of trained $B_1^+$ values (cf. Supplementary Fig. S10) – a behavior, which is similar to MIRACLE^27^.

Furthermore, this work is restricted to a single-component signal model by assuming that only a single $T_1$ and $T_2$ component at a single resonance frequency contributes to the acquired pc-bSSFP signal evolutions in tissues, thus not accounting for characteristic asymmetries in the frequency response of bSSFP. Those reflect anisotropies in tissue microstructure with a correlation to diffusion metrics, e.g. in WM^57–60^, or the sensitivity to chemical shift, which can be exploited for fat fraction mapping^61^. Comparable to MIRACLE, this results in an underestimation of $T_1$ and $T_2$ in brain tissues with respect to gold standard spin-echo-based reference methods^27,28^. The apparent rather flat $T_2$ contrast between white and gray matter of the proposed DNNs is consistent with MIRACLE (cf. for example Fig. 7) and typical for SSFP-based methods^28,62^.

In contrast to PINN, SVNN architectures are capable to identify nonlinear feature decodings, which cannot be modeled analytically. This can be exploited for the training of model-free SVNNs on in vivo data with independent ground truth MR measurements for each target parameter. However, supervised learning on in vivo data may be prone to input and target misalignment and necessitate prohibitively long scan times due to the need for ground truth data acquisition. Furthermore, the common ground in qMRI is dynamic and even current gold standard methods can be subject to various adverse instrumental factors related to the underlying sequence, hardware, or fitting routine, potentially leading to a quantification bias^63^.

Apart from analyzing the effectiveness of the proposed DNNs based on a direct comparison with conventional MIRACLE relaxometry, we demonstrated that the DNNs can conveniently be extended to utilize the full information of the complex-valued $F_n$ configurations (split into real and imaginary parts) instead of only taking magnitude data as input and can further be adapted to predict additional target parameters such as in this case $\Delta B_0$ to account for the off-resonance sensitivity introduced into the $F_n$ modes in case of undersampled phase cycles. The derived complex-based DNNs show promise for robust inference of $T_1$ and $T_2$ based on highly accelerated phase-cycled bSSFP imaging with as few as only 4 phase cycles, shortening the scan time by a factor of 3 (cf. Fig. 6 and Fig. 7). High undersampling in the phase cycle dimension comes along with increased noise levels in the parameter estimates, which could potentially introduce a bias and explain the residual differences observed in Figures Fig. 6 and Fig. 7 relative to the standard acquisition with 12 phase cycles.

Due to the ability to simulate, train, and infer tissue parameters in only a few seconds as demonstrated for the investigated magnitude-based DNNs in Fig. 8, in silico DNN training provides a cost-effective option and can easily be adapted to altered sequence parameters, new anatomical targets, or different field strengths, without requiring extensive MR data collection. Increasing the number of trainable parameters, which was essential in the training of the complex-based DNNs, resulted in the need for a larger number of epochs until convergence to reliable parameter estimation was reached, in particular in case of $T_1$ quantification with $PINN_{complex}$ (cf. Supplementary Fig. S8). The option for early stopping of the DNN training with a sufficiently high patience of 25 epochs ensured the acceptance of fluctuations in the validation loss and ultimate convergence to high-performing optima. Once trained, the magnitude-based DNNs were able to infer multi-parametric relaxation characteristics an order of magnitude faster than traditional iterative fitting as only a few matrix multiplications need to be performed (cf. Supplementary Fig. S9). The increased complexity in case of the complex-based DNNs yielded longer inference times on a single CPU thread, but still about 3 times faster in comparison to magnitude-based MIRACLE. The considerably shorter inference times of DNNs could become valuable in clinical settings, for example to make online derivation of quantitative maps feasible directly at the scanner console for real-time evaluation, or could facilitate studies necessitating the processing of large data cohorts. Future work may include the extension of the signal model employed for in silico DNN training to multi-compartment scenarios, the implementation of image-based architectures to benefit from anatomical information, and the application to pathological test data to validate the generalization performance in a clinical context.

Provided that the overall image quality in patients is similar as in our healthy test subjects and that the range of relaxation times in pathological tissue is covered by the simulated training data, we expect that the DNNs should yield reliable relaxometry inference when applied to pathological data since the training process is based on simulations and not on measurements. This should hold in particular for the PINNs, which have demonstrated to be highly independent of the underlying $T_1$ and $T_2$ training data distribution. Retraining may be needed in case of liquid-filled lesions or hematomas with prolonged relaxation times not covered by the training data distribution investigated in this work. The employed voxel-wise DNN fitting approach does not incorporate spatial information for parameter quantification, which reduces the risk to produce hallucinations in the relaxometry estimates in cases of brain abnormalities.

In conclusion, we have derived adaptable cost-effective deep learning frameworks for multi-parametric relaxometry based on pc-bSSFP data, which are characterized by rapid convergence during training, fast parameter inference times once training is concluded, and the ability to embed physical knowledge into the training process. By tailoring the underlying training data distribution to the target parameters of interest, superior performance to conventional fitting approaches could be achieved, especially in low-SNR scenarios, motivating further investigations at low field strengths. The adaptability of the proposed DNN relaxometry frameworks was successfully exploited for undersampling along the phase cycle dimension, enabling acceleration of the data acquisition by a factor of 3.

## Supplementary Information

Supplementary Information.

## Data Availability

The datasets analysed during the current study are available from the corresponding author on reasonable request.

## Code availability

The source code and trained models for this study are publicly available on https://github.com/s2quare/FastRelax-DL.
