# Supplementary material for: Flexible and cost-effective deep learning for accelerated multi-parametric relaxometry using phase-cycled bSSFP
Source: Sci Rep. 2025 Feb 9;15:4825. doi: 10.1038/s41598-025-88579-z (PMC11808094; doi:10.1038/s41598-025-88579-z)
Supplement: Supplementary file 1 — Supplementary Information. [file 41598_2025_88579_MOESM1_ESM.pdf]

Supplementary Information for:

Flexible and Cost-Effective Deep Learning for Accelerated Multi-Parametric Relaxometry using Phase-Cycled bSSFP

Florian Birk[1,2,*], Lucas Mahler[2], Julius Steiglechner[1,2], Qi Wang[2], Klaus Scheffler[1,2], and Rahel Heule[1,2,3]

[1]Department of Biomedical Magnetic Resonance, University of Tübingen, Tübingen, Germany
[2]High-Field Magnetic Resonance, Max Planck Institute for Biological Cybernetics, Tübingen, Germany
[3]Center for MR Research, University Children's Hospital, Zurich, Switzerland
[*]florian.birk@tuebingen.mpg.de

Supplementary Figures

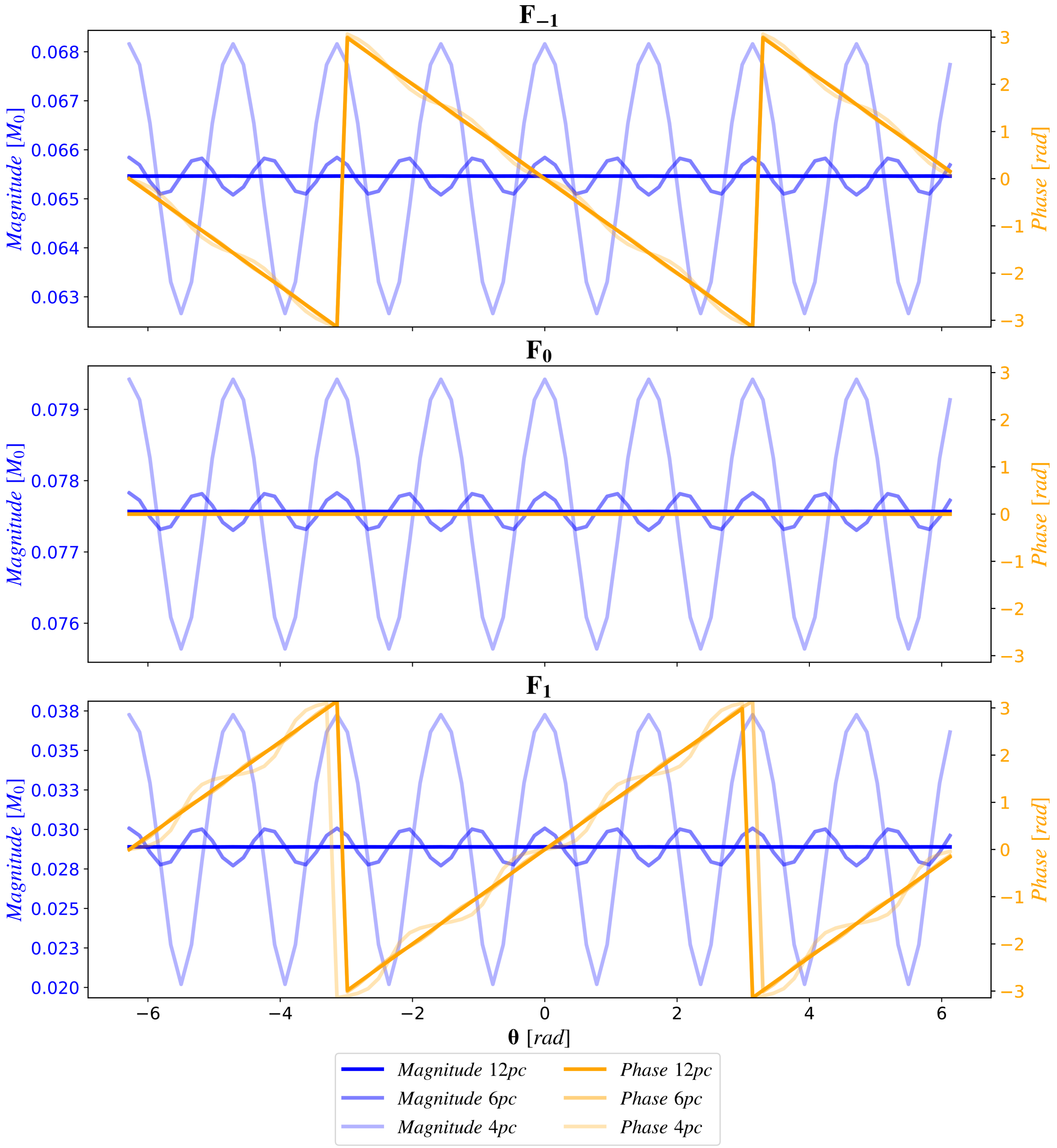

Figure S1. Effect of finite sampling of bSSFP phase cycles on the magnitude and phase of the three lowest-order SSFP configurations (F_1, F_0, F_1) derived via a discrete Fourier transform of the simulated series of bSSFP phase cycles. Simulations are performed for target white matter T_1 and T_2 values at 3 T[1]and off-resonance-related phase accumulation within TR (θ) ranging from -2π to $+2\pi$. It can be observed that a reduced number of phase cycles introduces off-resonance sensitivity due to aliasing in the F_n modes, which causes modulations of the magnitude and phase.

[1]Zhu, J., Klarhöfer, M., Santini, F., Scheffler, K. & Bieri, O. Relaxation Measurements in Brain Tissue at Field Strengths Between 0.35T and 9.4T. In ISMRM-ESMRMB 2014 (Milano, Italy, 2014).

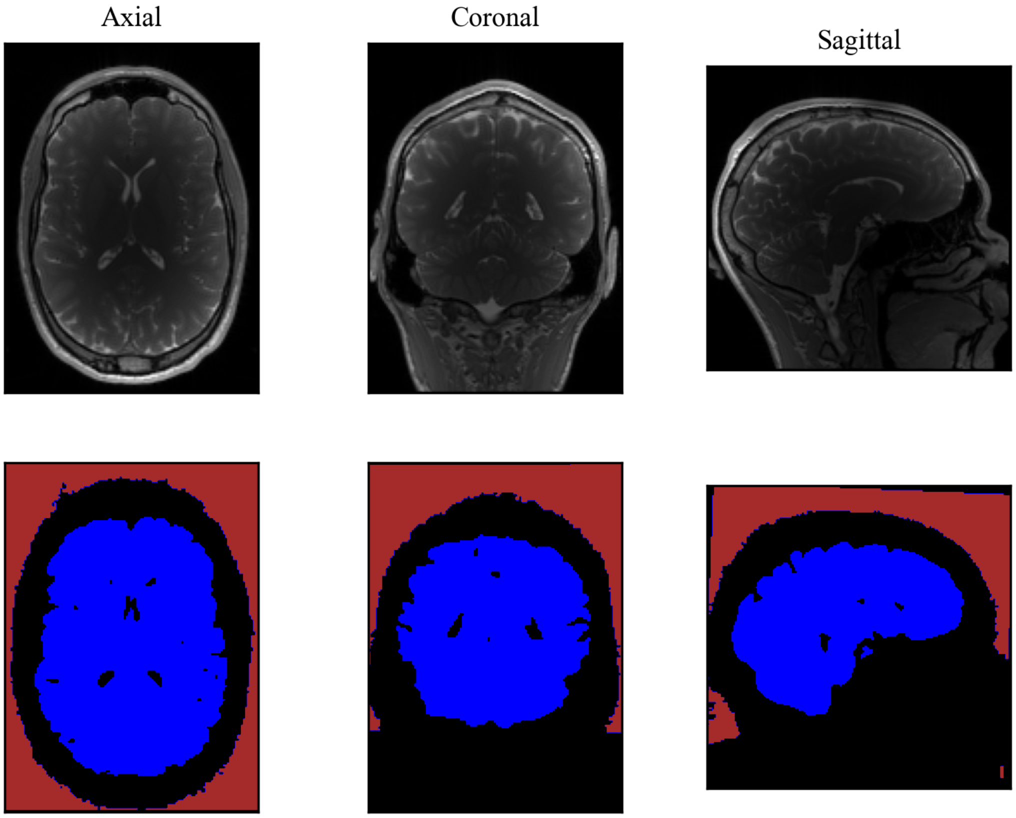

Figure S2. Representative axial, coronal, and sagittal slices of one of the three subjects used for SNR determination based on the acquired in vivo pc-bSSFP data. The magnitude of the complex sum (F_0) (first row) was used for calculating the average signal (S) within the brain mask (blue mask in second row). The noise (N) was calculated as the standard deviation within the background mask (red mask in second row). The final $SNR = S/N$ was pooled over all three subjects.

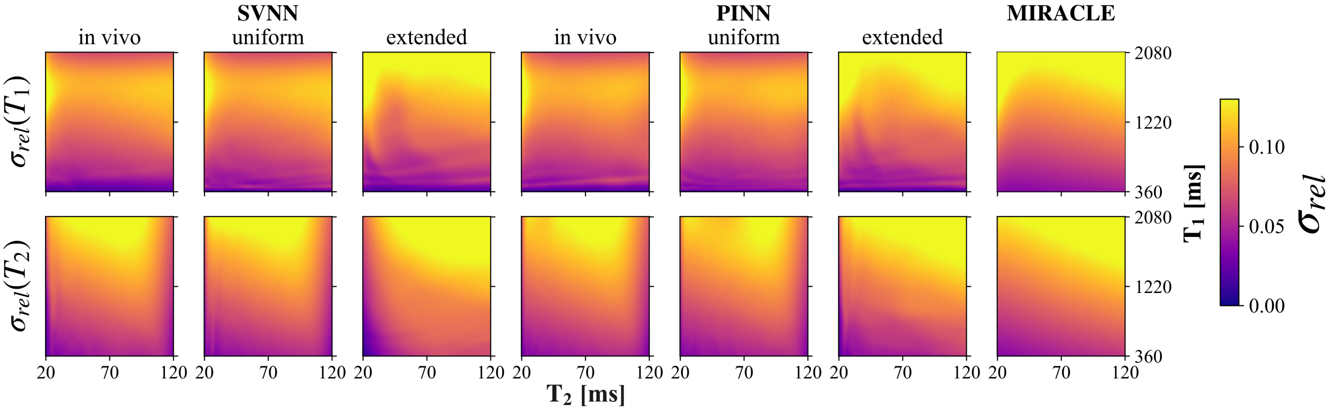

Figure S3. Influence of training data distribution on precision of investigated standard magnitude-based DNNs versus MIRACLE in silico. The precision of T_1 and T_2 quantification is evaluated by MC sampling with an **SNR level of 25**, matched to the in vivo data, applied to an in silico linear test grid with T_1 in the range 360 to 2080 ms and T_2 in the range 20 to 120 ms. The relative standard deviation $\sigma_{rel} = \sigma_{\text{MC}}/\mu_{\text{MC}}$, with μ_{MC} and σ_{MC} corresponding to the mean and standard deviation of the MC simulation is plotted for all three frameworks (SVNN, PINN, MIRACLE) and in case of the DNNs for all three trained data distributions. All DNNs were trained without additional noise applied to the training data (SNR = inf).

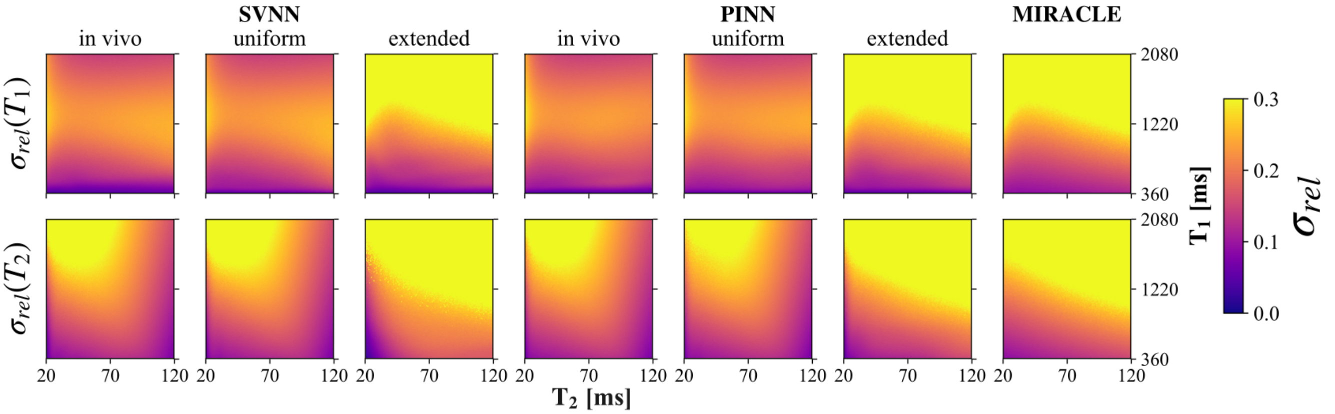

Figure S4. Influence of training data distribution on precision of investigated standard magnitude-based DNNs versus MIRACLE in silico. The precision of T_1 and T_2 quantification is evaluated by MC sampling with an **SNR level of 10**, applied to an in silico linear test grid with T_1 in the range 360 to 2080 ms and T_2 in the range 20 to 120 ms. The relative standard deviation $\sigma_{rel} = \sigma_{\text{MC}}/\mu_{\text{MC}}$, with μ_{MC} and σ_{MC} corresponding to the mean and standard deviation of the MC simulation is plotted for all three frameworks (SVNN, PINN, MIRACLE) and in case of the DNNs for all three trained data distributions. All DNNs were trained without additional noise applied to the training data (SNR = inf).

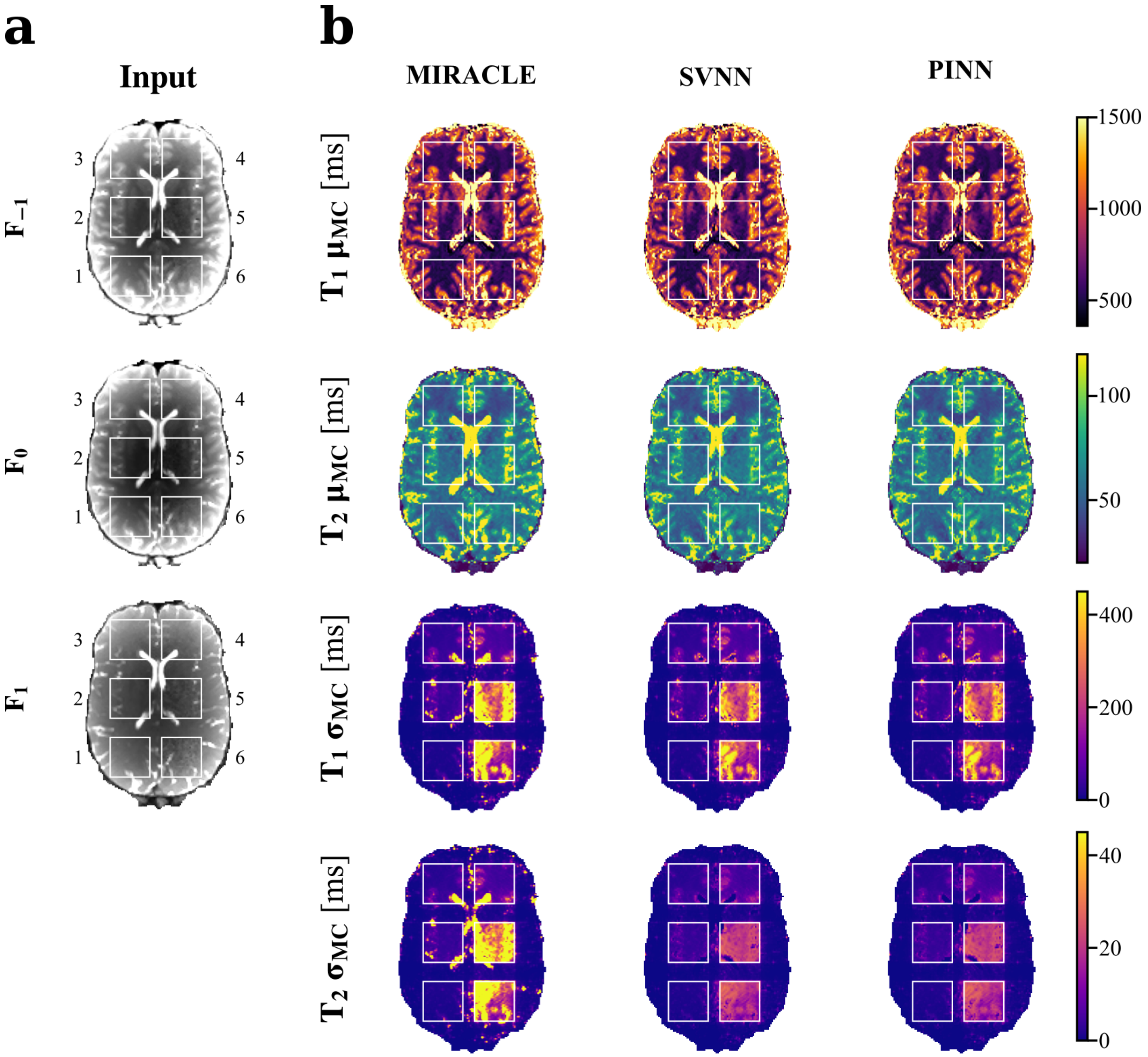

Figure S5. Robustness in the presence of noise-corrupted in vivo test data of standard magnitude-based SVNN and PINN versus MIRACLE, illustrated for a representative axial slice of an unseen test subject. (**a**) The multi-contrast input for quantification of the relaxation parameters, i.e. the magnitude of F_{-1}, F_0, and F_1, for an individual MC noise sample. (**b**) The mean (μ_{MC}, rows 1+2) and standard deviation (σ_{MC}, rows 3+4) of the in vivo MC parameter predictions. The displayed results refer to DNNs trained on in silico data with the **uniform distribution** and no additional noise (SNR = inf). In addition to the existing noise of the in vivo test data, noise sampled from a Gaussian distribution with six different standard deviations ($\eta \in \{1,2,4,8,12,16\}$ and respective in vivo SNRs $\in \{18,14,11,7,5,4\}$) was added to the real and imaginary parts of the pc-bSSFP data in six different rectangular ROIs, labeled 1-6 in the order of increasing noise levels.

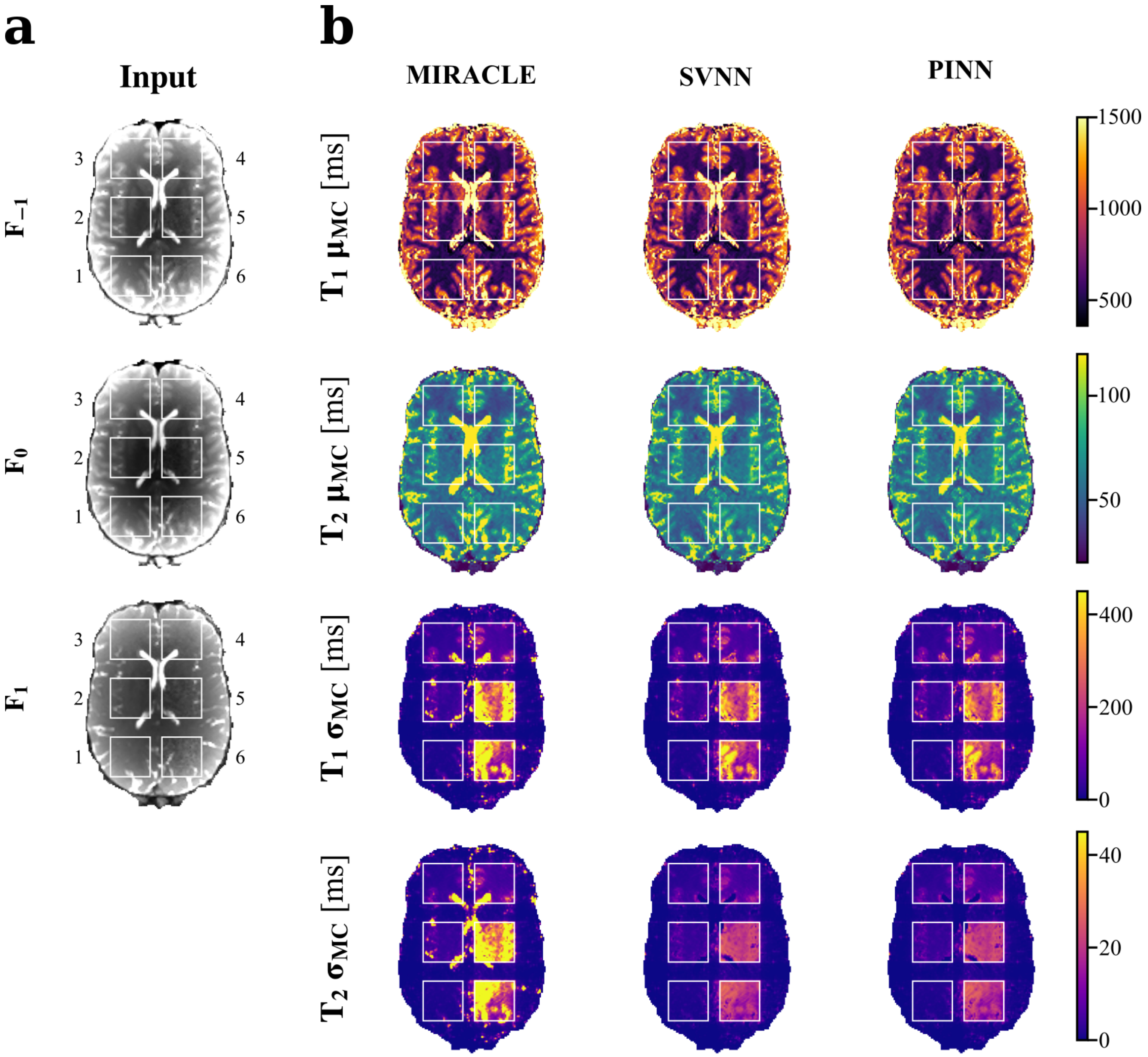

Figure S6. Robustness in the presence of noise-corrupted in vivo test data of standard magnitude-based SVNN and PINN versus MIRACLE, illustrated for a representative axial slice of an unseen test subject. (**a**) The multi-contrast input for quantification of the relaxation parameters, i.e. the magnitude of F_{-1}, F_0, and F_1, for an individual MC noise sample. (**b**) The mean (μ_{MC}, rows 1+2) and standard deviation (σ_{MC}, rows 3+4) of the in vivo MC parameter predictions. The displayed results refer to DNNs trained on in silico data with the **in vivo distribution** and no additional noise (SNR = inf). In addition to the existing noise of the in vivo test data, noise sampled from a Gaussian distribution with six different standard deviations ($\eta \in \{1, 2, 4, 8, 12, 16\}$ and respective in vivo SNRs $\in \{18, 14, 11, 7, 5, 4\}$) was added to the real and imaginary parts of the pc-bSSFP data in six different rectangular ROIs, labeled 1-6 in the order of increasing noise levels.

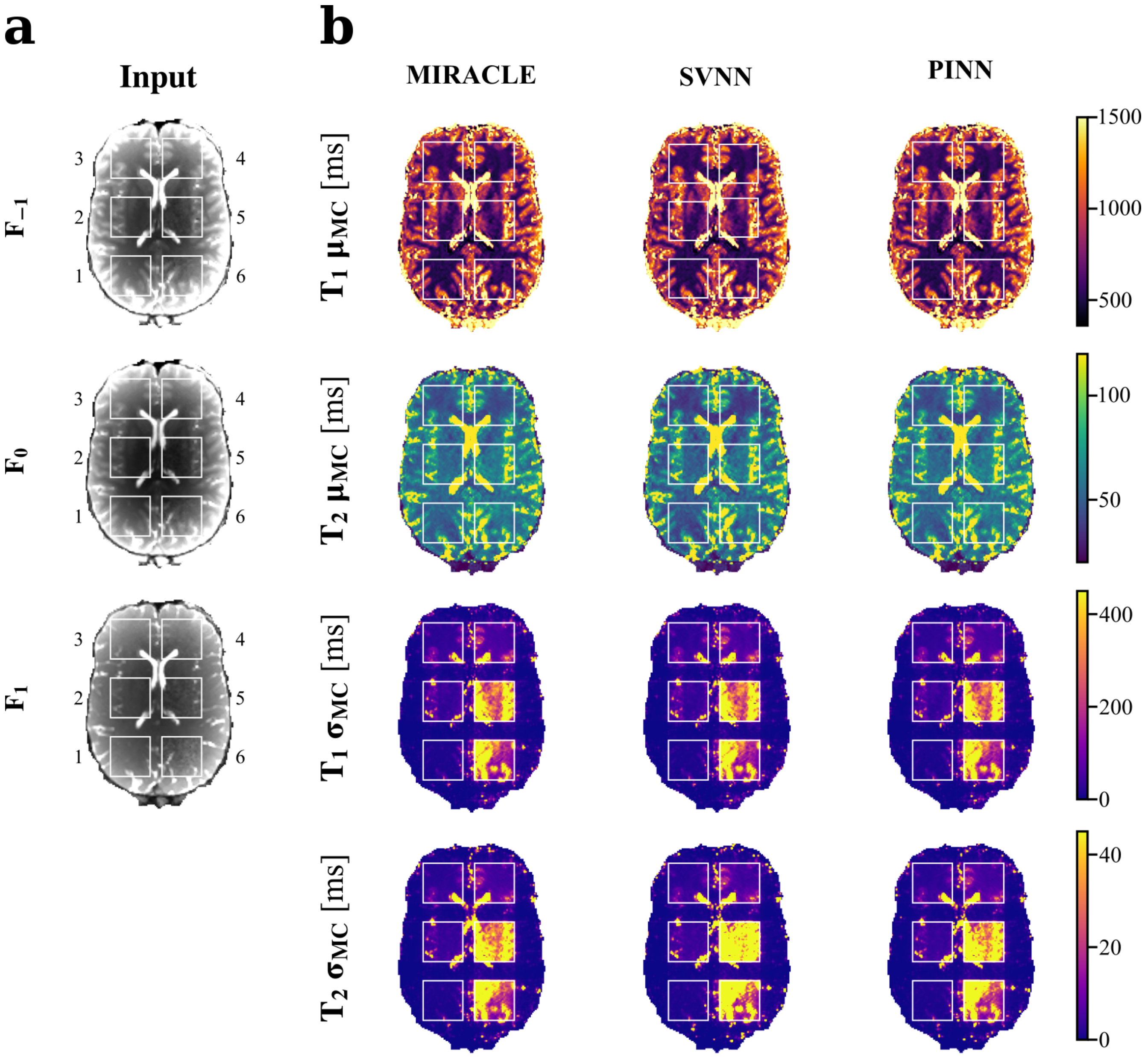

Figure S7. Robustness in the presence of noise-corrupted in vivo test data of standard magnitude-based SVNN and PINN versus MIRACLE, illustrated for a representative axial slice of an unseen test subject. (**a**) The multi-contrast input for quantification of the relaxation parameters, i.e. the magnitude of F_{-1}, F_0, and F_1, for an individual MC noise sample. (**b**) The mean (μ_{MC}, rows 1+2) and standard deviation (σ_{MC}, rows 3+4) of the in vivo MC parameter predictions. The displayed results refer to DNNs trained on in silico data with the **uniform extended distribution** and no additional noise (SNR = inf). In addition to the existing noise of the in vivo test data, noise sampled from a Gaussian distribution with six different standard deviations ($\eta \in \{1,2,4,8,12,16\}$ and respective in vivo SNRs $\in \{18,14,11,7,5,4\}$) was added to the real and imaginary parts of the pc-bSSFP data in six different rectangular ROIs, labeled 1-6 in the order of increasing noise levels.

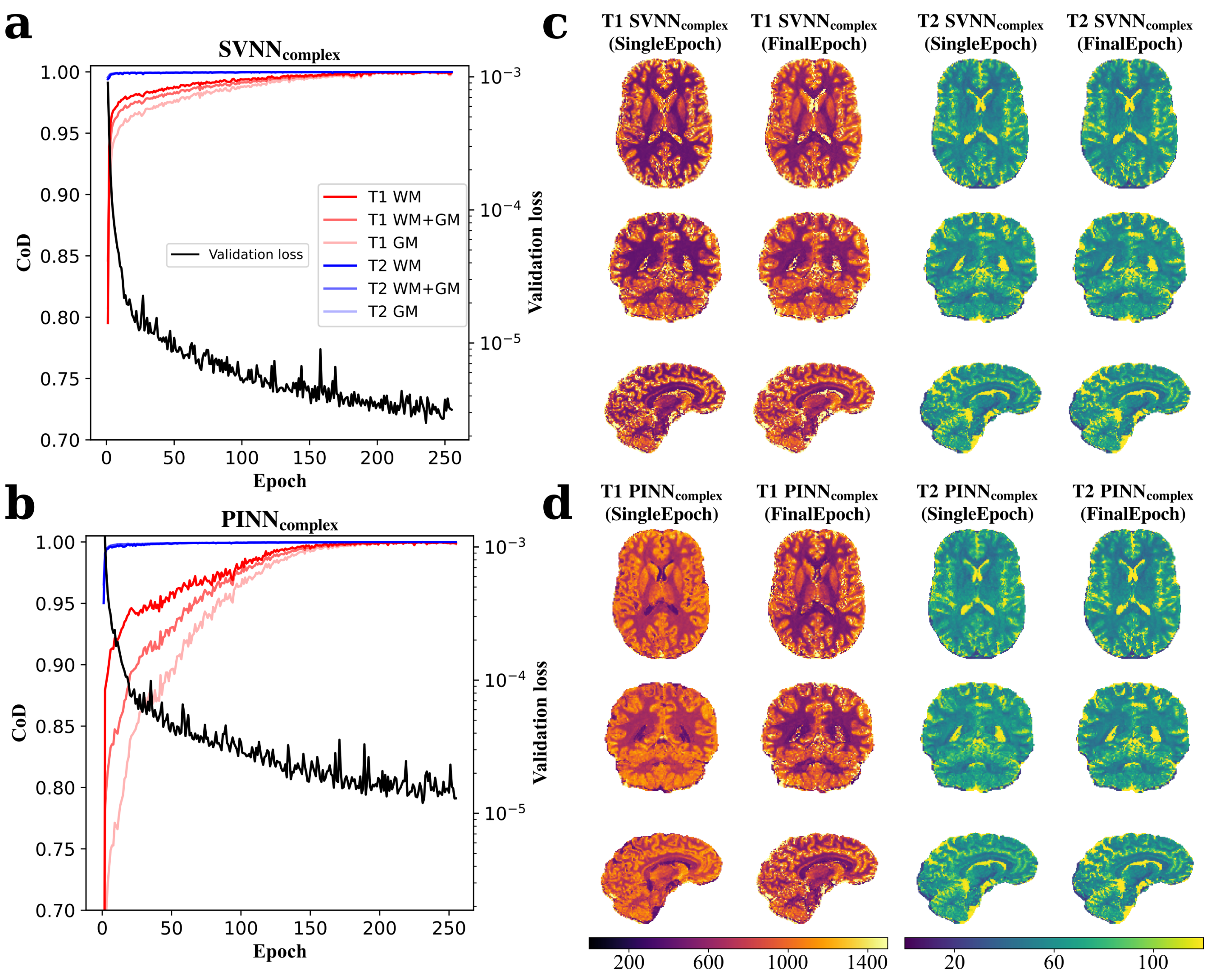

Figure S8. Efficiency of $DNN_{complex}$ inverse signal model learning versus epochs, corroborated by representative relaxation time maps of single-epoch in vivo whole-brain inference. The CoD during $SVNN_{complex}$ (**a**) and $PINN_{complex}$ (**b**) training is calculated for each epoch with respect to the final-epoch model and plotted versus epochs for in vivo T_1 (red) and T_2 (blue) predictions in whole-brain WM, GM, and WM+GM tissue masks of an unseen test subject. Additionally, the validation loss for both $DNN_{complex}$ frameworks is shown in black on a logarithmic scale. The employed $DNNs_{complex}$ were trained on the in silico uniform noise-free data distribution. Note that the final validation loss of the $SVNN_{complex}$ framework is on the order of one magnitude lower than the one of the $PINN_{complex}$ framework due to the different definitions of the loss functions and embedding of physical constraints for the $PINN_{complex}$. Corresponding representative axial, coronal, and sagittal slices of in vivo whole-brain T_1 and T_2 single-echo versus final-epoch predictions of an unseen test subject are shown for $SVNN_{complex}$ (**c**) and $PINN_{complex}$ (**d**).

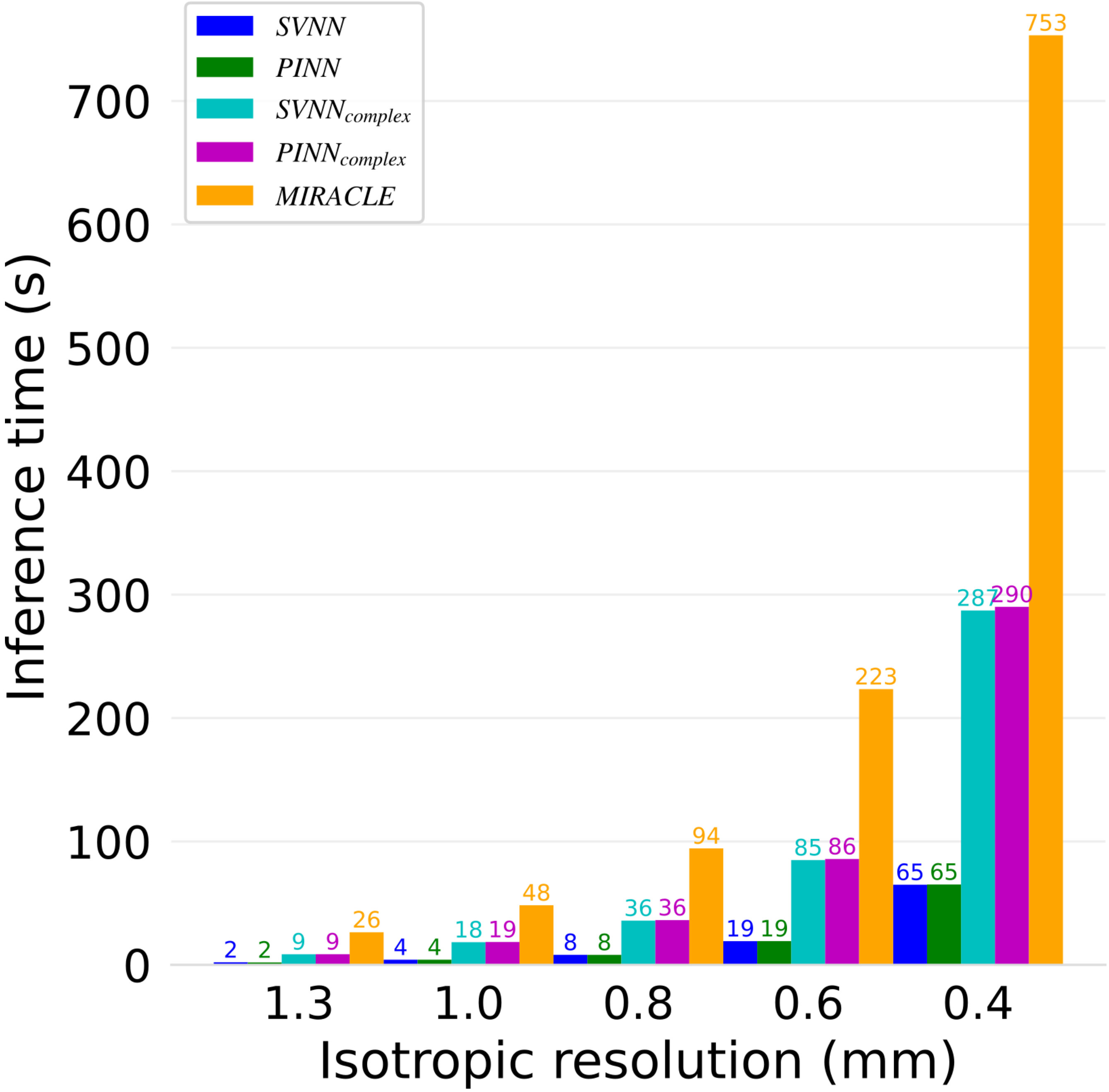

Figure S9. Whole-brain relaxometry inference times for different isotropic resolutions of the input data. The inference times in seconds of each multi-parametric relaxometry framework (SVNN: blue, PINN: green, $SVNN_{complex}$: cyan, $PINN_{complex}$: purple, MIRACLE: orange) are calculated for the whole-brain input data of a test subject interpolated to different isotropic resolutions of 1.3, 1.0, 0.8, 0.6 and 0.4 mm. Inference is performed using a single CPU thread (Intel(R) Xeon(R) W-2255 CPU @ 3.70GHz, 62.5 GB RAM).

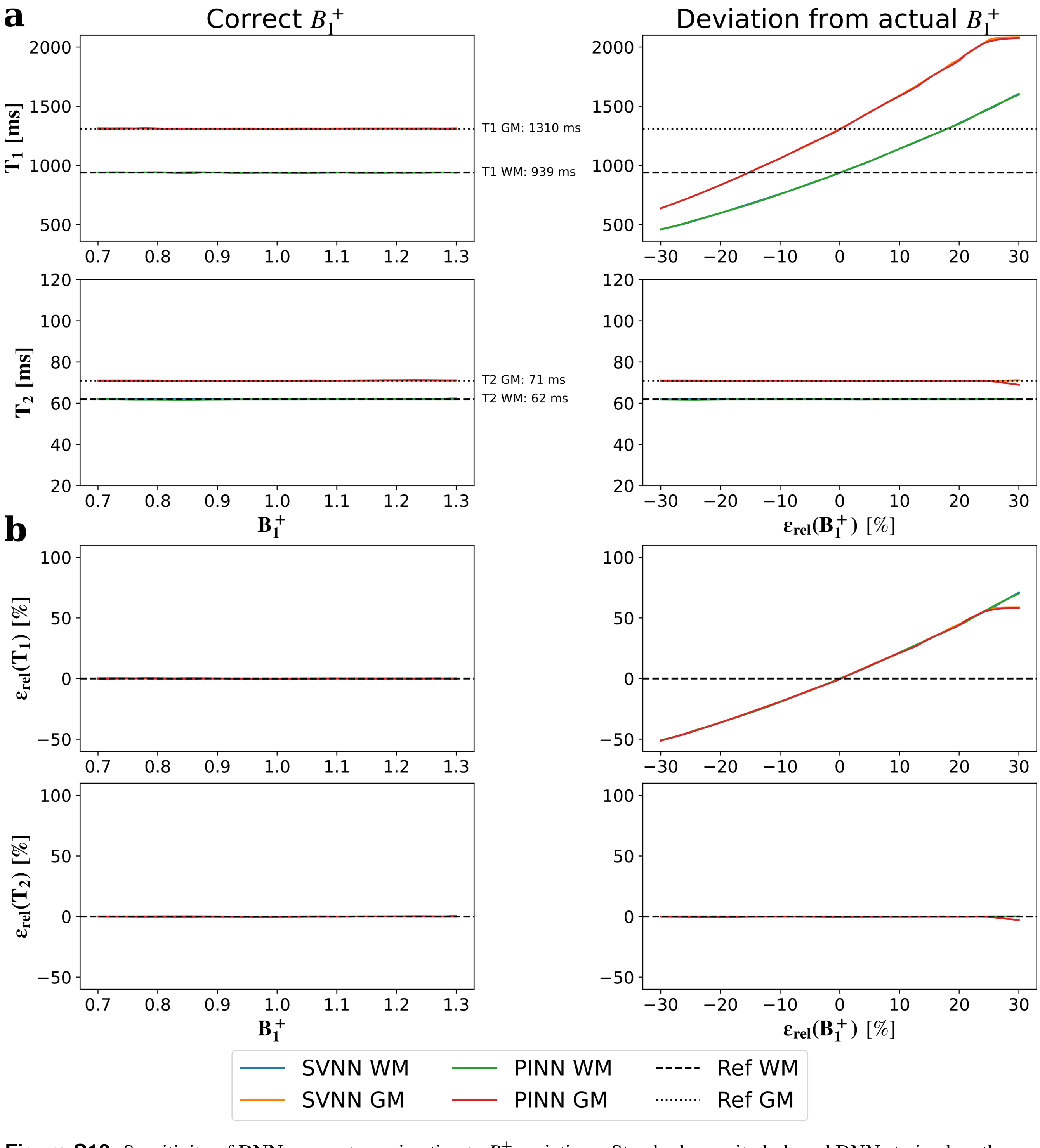

Figure S10. Sensitivity of DNN parameter estimation to B_1^+ variations. Standard magnitude-based DNNs trained on the noise-free uniform data distribution are assessed versus the actual B_1^+ in the range of trained values ($B_{1,act}^+$ = [0.7, 1.3]). In the left column, it is assumed that the externally measured B_1^+, which is used as input for the DNNs, corresponds to the actual B_1^+, which is plotted on the x-axis. In the right column, it is assumed that the externally measured B_1^+ is equal to 1.0 with the relative deviations from the actual B_1^+ displayed on the x-axis, calculated as $\varepsilon_{rel}(B_1^+) = (B_{1,act}^+ - B_{1,meas}^+)/B_{1,meas}^+ \cdot 100$. (**a**) Absolute prediction of target white matter (WM) and gray matter (GM) T_1 and T_2 values at 3 T[1] and (**b**) corresponding relative error between the parameter predictions $\hat{T}_i$ and the simulated ground truth value T_i, $\varepsilon_{rel}(T_i) = (\hat{T}_i - T_i)/T_i \cdot 100$ with i = 1, 2, are shown for the correct B_1^+ (left column) and $\varepsilon_{rel}(B_1^+)$ (right column).
